# A review on machine learning approaches and trends in drug discovery

**DOI:** 10.1016/j.csbj.2021.08.011

**Published:** 2021-08-12

**Authors:** Paula Carracedo-Reboredo, Jose Liñares-Blanco, Nereida Rodríguez-Fernández, Francisco Cedrón, Francisco J. Novoa, Adrian Carballal, Victor Maojo, Alejandro Pazos, Carlos Fernandez-Lozano

**Affiliations:** a Department of Computer Science and Information Technologies, Faculty of Computer Science, Universidade da Coruna, Campus Elviña s/n, A Coruña 15071, Spain; b CITIC-Research Center of Information and Communication Technologies, Universidade da Coruna, A Coruña 15071, Spain; c Department of Computer Science and Information Technologies, Faculty of Communication Science, Universidade da Coruna, Campus Elviña s/n, A Coruña 15071, Spain; d Biomedical Informatics Group, Artificial Intelligence Department, Polytechnic University of Madrid, Calle de los Ciruelos, Boadilla del Monte, Madrid 28660, Spain; e Grupo de Redes de Neuronas Artificiales y Sistemas Adaptativos. Imagen Médica y Diagnóstico Radiológico (RNASA-IMEDIR), Complexo Hospitalario Universitario de A Coruña (CHUAC), SERGAS, Universidade da Coruña, Instituto de Investigación Biomédica de A Coruña (INIBIC), A Coruña, Spain

**Keywords:** Machine Learning, Drug Discovery, Cheminformatics, QSAR, Molecular Descriptors, Deep Learning, ML, Machine Learning, AI, Artificial Intelligence, SMILES, simplified molecular-input line-entry system, DNA, Deoxyribonucleic acid, RNA, Ribonucleic Acid, PCA, Principal Component Analyisis, t-SNE, t-Distributed Stochastic Neighbor Embedding, FS, Feature Selection, CV, Cross Validation, QSAR, Quantitative structure–activity relationship, MD, Molecular Descriptors, FP, Fringerprints, ECFP, Extended Connectivity Fingerprints, MACCS, Molecular ACCess System, APFP, Atom Pairs 2d FingerPrint, CDK, Chemical Development Kit, SVM, Support Vector Machines, ANN, Artificial Neural Networks, NB, Naive Bayes, FNN, Fully Connected Neural Networks, CNN, Convolutional Neural Networks, GNN, Graph Neural Networks, GCN, Graph Convolutional Networks, ADMET, Absorption, distribution, metabolism, elimination and toxicity, ADR, Adverse Drug Reaction, CPI, Compound-protein interaction, CNS, Central Nervous System, BBB, Blood–Brain barrier, KEGG, Kyoto Encyclopedia of Genes and Genomes, WHO, World Health Organization, AUC, Area under the Curve, GEO, Gene Expression Omnibus, FDA, Food and Drug Administration, MKL, Multiple Kernel Learning, OOB, Out of Bag, TCGA, The Cancer Genome Atlas, GO, Gene Ontology, MCC, Matthews correlation coefficient, RF, Random Forest, DL, Deep Learning

## Abstract

Drug discovery aims at finding new compounds with specific chemical properties for the treatment of diseases. In the last years, the approach used in this search presents an important component in computer science with the skyrocketing of machine learning techniques due to its democratization. With the objectives set by the Precision Medicine initiative and the new challenges generated, it is necessary to establish robust, standard and reproducible computational methodologies to achieve the objectives set. Currently, predictive models based on Machine Learning have gained great importance in the step prior to preclinical studies. This stage manages to drastically reduce costs and research times in the discovery of new drugs. This review article focuses on how these new methodologies are being used in recent years of research. Analyzing the state of the art in this field will give us an idea of where cheminformatics will be developed in the short term, the limitations it presents and the positive results it has achieved. This review will focus mainly on the methods used to model the molecular data, as well as the biological problems addressed and the Machine Learning algorithms used for drug discovery in recent years.

## Highlights

• Machine Learning in drug discovery has greatly benefited the pharmaceutical industry.
• Application of machine algorithms must entail a robust design in real clinical tasks.
• Trending machine learning algorithms in drug design: NB, SVM, RF and ANN.

## Graphical abstract

**Figure:**
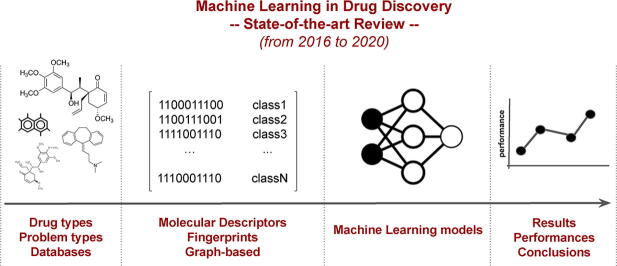

## 1 Introduction

According to the Precision Medicine Initiative, precision medicine is “an emerging approach for disease treatment and prevention that takes into account individual variability in genes, environment and lifestyle for each person” [1]. This new approach allows physicians and researchers to increase accuracy in predicting disease treatment and prevention strategies that will work for particular groups of people. This approach contrasts with the “one-size-fits-all” approach, more widely used until relatively recently, in which the strategies mentioned above are developed with the average person in mind, regardless of differences between individuals.

The opportunity for the creation of new treatments offered by precision medicine generates at the same time great difficulties in the development of new methodologies. For this reason, in recent years a large amount of biomedical data has been generated, coming from very diverse sources: from small individual laboratories to large international initiatives. These data, known mostly as omic data (genomic, proteomic, metabolomic, pharmacogenomic, etc.), are an inexhaustible source of information for the scientific community, which allows stratifying patients, obtaining specific diagnoses or generating new treatments [2].

Diagnostic tests are frequently performed in some disease areas, as they allow immediate identification of the most effective treatment for a specific patient through a specific molecular analysis. With this, the practice of trial and error medicine, which is often frustrating and considerably more expensive, is often avoided. In addition, drugs created from these molecular characteristics usually improve treatment results and reduce side effects. One of the most common examples can be found in the treatment of patients with breast cancer. A significant percentage of patients with this type of tumor are characterized by overexpression of human epidermal growth factor receptor 2 (HER2). For these patients, treatment with the drug trastuzumab (Herceptin) in addition to chemotherapy treatment can reduce the risk of recurrence to more than 50% [3].

On the other hand, there are also the so-called pharmacogenomic tests that provide assistance in making decisions related to the drug and the dose formulated for each patient. These decisions are based on the genomic profiles of the patients, so that they can metabolize certain drugs in different ways according to their genetics, thus causing adverse reactions. These reactions are related to variants in the genes that encode drug metabolizing enzymes, such as cytochrome P450 (CYP450). Pharmacogenomic testing can contribute to the safe and effective application of drugs in many different areas of health, including heart disease, cancer adjunctive therapy, psychiatry, HIV and other infectious diseases, dermatology, etc.

The greatest complexity in drug discovery for certain molecular targets and/or patient subgroups is found in the process itself and in the strict regulations presented by the regulatory bodies. Currently, the discovery and development of new drugs is still a long and extremely costly process. The average time period for the development of a new drug is between 10 and 15 years of research and testing. The large number of existing molecules with the capacity to be tested as new drugs makes their study in wet lab experiments practically impossible. However, in the last decade, the evolution of the Information and Communication Technologies, as well as the increase of the available computational capacity, has given way to new methodologies *in silico* for the screening of extensive drug libraries. This step prior to preclinical studies reduces the economic cost and increases the space for searching for new drugs. In this context, Machine Learning (ML) techniques have gained a great prominence in the pharmaceutical industry, offering the ability to accelerate and automate the analysis of the large amount of data currently available.

The ML is a branch of Artificial Intelligence (AI) that aims to develop and apply computer algorithms that learn from raw, unprocessed data, in order to later perform a specific task. The main tasks performed by the AI algorithms are classification, regression, clustering or pattern recognition within a large data set. There is a great variety of ML methods that have been used in the pharmaceutical industry for the prediction of new molecular characteristics, biological activities, interactions and adverse effects of drugs. Some examples of these methods are Naive Bayes, Support Vector Machines, Random Forest and, more recently, Deep Neural Networks [4], [5], [6], [7], [8], [9], [10].

In order to study the state of the art in this field, this work has been designed and developed. It gathers the most relevant publications of the last five years in the use of ML techniques for early drug discovery. Next, the works identified in this study are presented in different sections, analyzing with special interest the descriptors used, the biological problem to be solved and the ML algorithm used.

## 2 Standard machine learning methodology

**Fig. 1:**
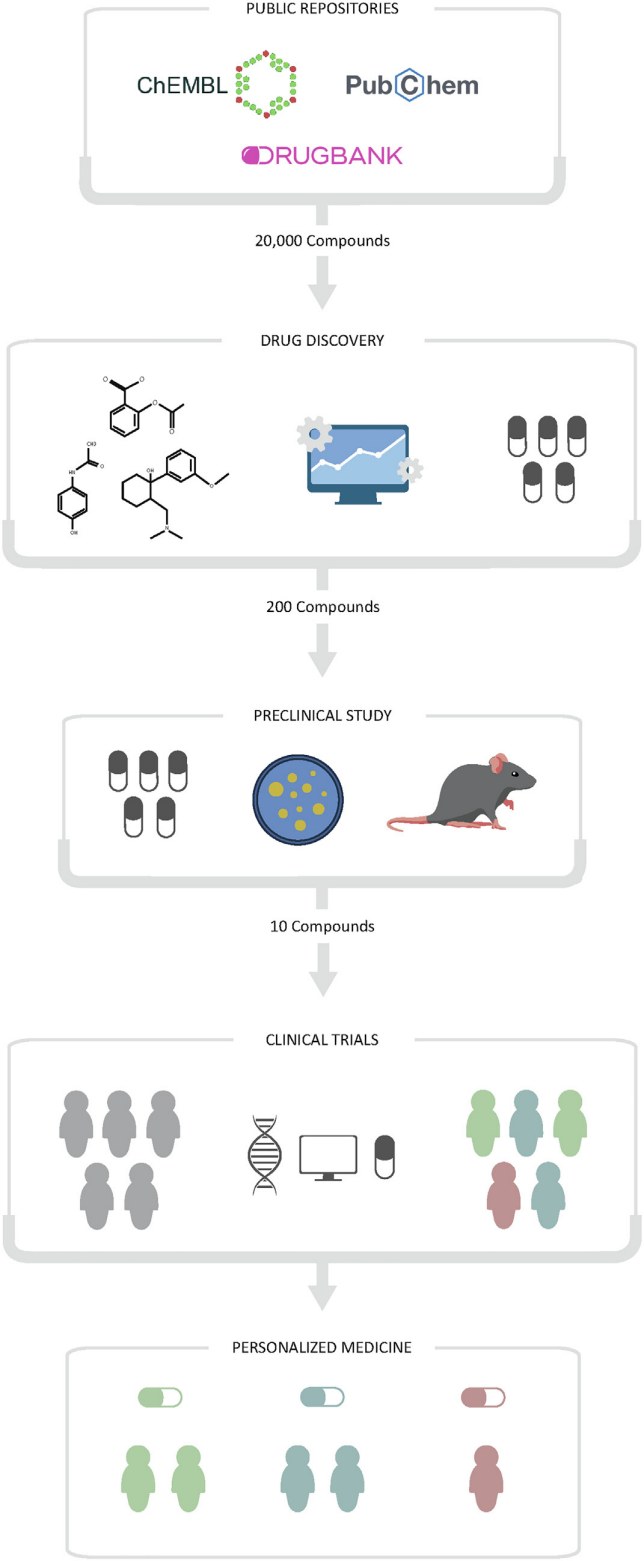
Stages in the discovery of new drugs in the context of precision medicine.

The design of the experimental phase is a crucial step in the field of Computational Intelligence and especially in ML. For this, it is essential to first define the methodology to be implemented.Fig. 1.

The application of an ML methodology must be transversal in any field of research [11], even if all fields share certain steps in the experimental design. Specifically, in the ML methodology applied in drug discovery we can differentiate the following steps: 1) data collection; 2) generation of mathematical descriptors; 3) search for the best subset of variables; 4) model training; 5) model validation.

**Fig. 2:**
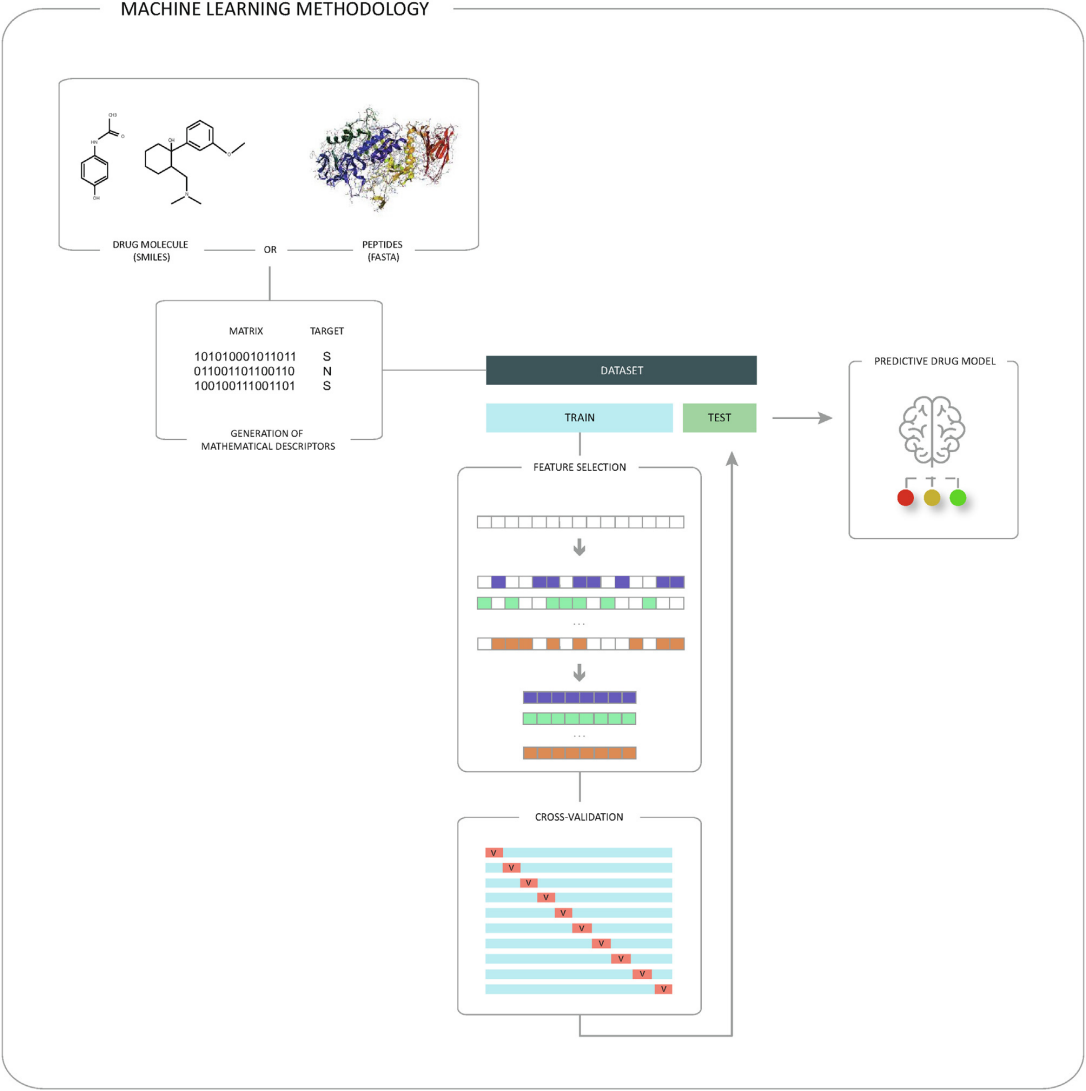
Machine Learning methodology commonly used for drug discovery.

**Fig. 3:**
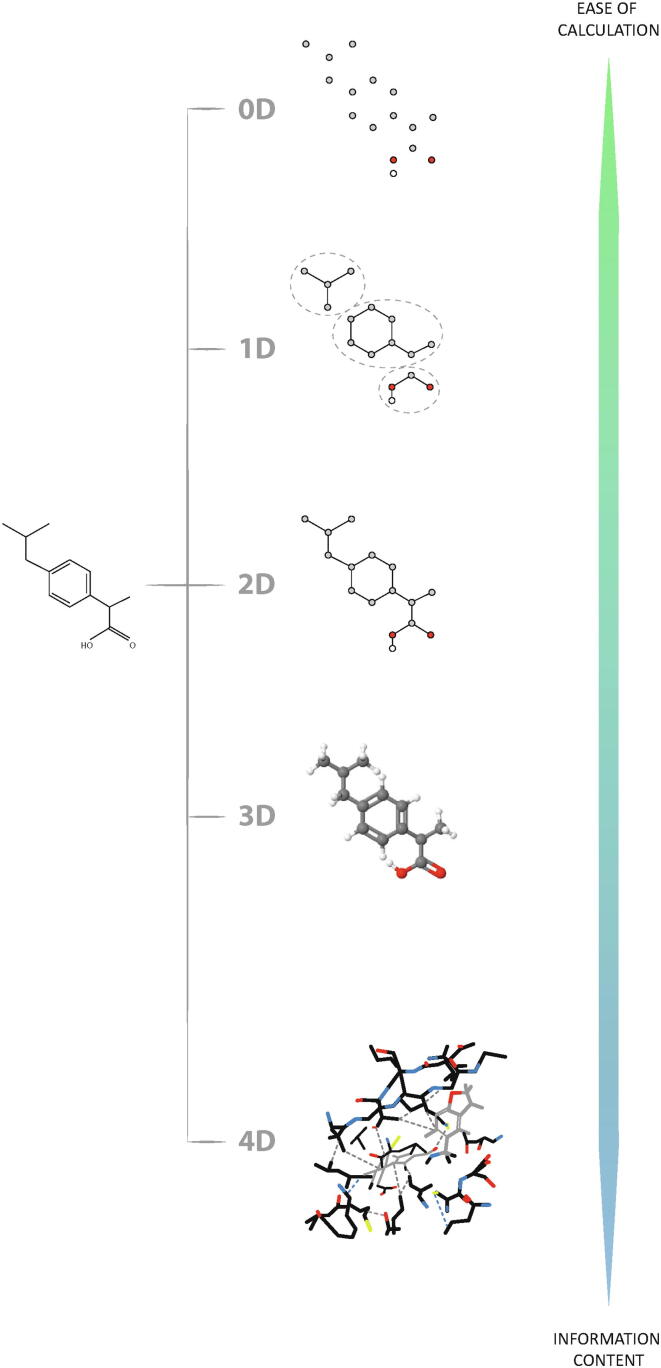
Representation of the information coded by the different molecular descriptors according to their dimensions.

In Fig. 2, a diagram of the Machine Learning methodology commonly used for drug discovery can be observed Fig. 3.

The first step is to obtain the data set, which must have certain characteristics. In addition to physical–chemical characteristics that help absorption, specificity and low toxicity, it must also have characteristics that allow it to be easily produced and handled in the laboratory. This is because the pharmaceutical industry does not employ large proteins or extremely complex molecules. The main compounds it usually works on include small molecules and peptides. In order to simplify the handling and analysis of these compounds, the SMILES and FASTA formats are used to represent the sequence and structure of small molecules and peptides, respectively.

Currently, there are numerous public repositories 1 that store a large amount of useful data for the field of drug discovery, such as DrugBank [12], PubChem [13], ChEMBL [14] or ZINC [15].

**Table 1:** Top public repositories with chemistry used in Machine Learning model training. Table shows number of availables compounds in each repository and its usability.

Database	**No Compounds**	**Usabillity**	**Link**	**Reference**
DrugBank	14 K	Drug Discovery	https://go.drugbank.com/	[12]
PubChem	110 M	Computational Chemistry	https://pubchem.ncbi.nlm.nih.gov/	[13]
ChEMBL	2.1 M	Drug Discovery	https://www.ebi.ac.uk/chembl/	[14]
ZINC	750 M	Virtual Screening	https://zinc.docking.org/	[15]

New sequencing technologies have made great progress in generating sequence data (DNA, RNA, proteins, small molecules, etc.). The sequences of compounds are the starting point in drug discovery, although few mathematical models are capable of generating predictions based solely on these sequences. In order to perform the prediction, it is necessary to convert the sequences into matrices that can be subsequently addressed by ML algorithms (matrix in Fig. 2). The labelling of the different compounds is also important (target in Fig. 2). Although there are some ML models that do not need labelling, it is common in the field of drug discovery to use supervised learning models. In this case, the labelling defined by the researchers will be essential in the experimental process.

With the generation of the mathematical descriptors, a set of data is obtained which the ML model can process. This dataset is divided into two subsets: one with a higher percentage of data, dedicated to training the model (represented by the blue colour in Fig. 2 smaller one dedicated to testing the model (represented by the green colour in Fig. 2).

Within the training set, the search for the best subset of variables is carried out, with the right and necessary information. Normally, during the generation of mathematical descriptors, a large number of numerical variables are presented. The main objective of this process is to reduce as much as possible the useless or redundant variables. To this end, there are different techniques such as PCA, t-SNE, FS, Autoencoder, etc. FS techniques obtain a subgroup of features belonging to the original set, which does not modify the content of the variables. This provides a justification that is understandable at a biological level and that is why a large majority of researchers use these techniques in their experimental designs [16].

Once the optimal subset of variables has been located, the model is trained. First, the algorithms and their parameters must be selected. These must be chosen carefully to ensure that they are appropriate to the problem in question and the amount and type of data available. Then, different runs of the experiment are performed with the training data. Excessive training should be avoided to ensure the validity of the model with unknown data. The use of techniques such as cross-validation (CV) is common in these cases. The CV allows measuring the degree of generalization of the model during the training phase, evaluating its performance and estimating the performance with unknown data. In each execution of the experiment, the original data set is divided again into two subsets: the training set and the validation set. In the Fig. 2 you can see the development of the CV technique with 10 runs. For each of these runs, the blue set corresponds to the training set and the red set to the validation set.

The ultimate goal of the CV process is to select the best combination of parameters for each technique. From these parameters the performance of each model is measured. The best model is the one that achieves the highest performance value with the lowest total cost.

Finally, the test set extracted from the original set is recovered (represented by the green colour in the Fig. 2 and a final validation process of the best model resulting from the CV process is carried out. If the validation results are statistically significant [17], it can be said that a new predictive drug model has been created.

Machine learning techniques have been used in many fields, and the number of published papers has increased especially in recent years. However, few of the machine learning publications related to drug development are found on open access websites. To facilitate this understanding, works such as [18], [19] provide an overview of machine learning techniques and the current state of applications for drug discovery in both academic and industrial settings.

## 3 The importance of input data in Machine Learning predictions

A critical step in the training of the model depends on the representation of the molecules by descriptors that are capable of capturing their properties and structural characteristics. Hundreds of molecular descriptors have been reported in the literature ranging from simple properties of the molecules to elaborate three-dimensional and complex molecular fingerprint formulations, stored in vectors of hundreds and/or thousands of elements.

### 3.1 Quantitative structure–activity relationship

Under the premises “the structure of a molecule defines its biological activity” and “structurally similar molecules have a similar biological activity”, the models of quantitative relationship between structure and activity (QSAR), which numerically relate the chemical structures of the molecules with their biological activity, allow, through mathematical systems, to predict the physicochemical and biological fate properties that a new compound will have from the knowledge of its chemical structure and from existing experimental studies.

QSAR models integrate computer and statistical techniques in order to make a theoretical prediction of biological activity that allows the theoretical design of possible future new drugs, avoiding the trial and error process of organic synthesis. As it is a science that exists only in a virtual environment, it allows dispensing with certain resources such as equipment, instruments, materials and laboratory staff. With a focus on the relationships between chemical structure and biological activity, the design of candidates for new drugs is much cheaper and faster. Modeling studies such as QSAR is one of the most effective methods to perform compound prediction when there is a lack of adequate experimental data and facilities [20].

To carry out a QSAR study, three types of information are needed [21]:

1. **Molecular structure** of different compounds with a common mechanism of action
2. **Biological activity data** of each of the ligands included in the study.
3. **Physicochemical properties**, which are described from a set of numerical variables, obtained from the molecular structure virtually generated by computational techniques.

In the prospective type, the results in the form of equation or QSAR model allow predicting the biological activity of compounds not yet synthesized that are generated virtually in a short time, but must share structural characteristics of the ligands included in the study not to leave the rules or chemical pattern or range of values of the descriptors. The other type, the retrospective analyzes the already existing molecules (those of synthesis and bioassays) to understand their non-obvious interrelations between structures and biological activities. The preparation of the input data is the most crucial step since the result is obtained in an automated way and only depends on the input.

The QSAR methodology is interdisciplinary, so it receives information from Organic Chemistry and Pharmacology. The way in which QSAR rewards this situation and that constitutes the objective of this methodology, is through the directed design of ligands that do not yet exist, but through the generated equations have shown a high probability of pharmacological success because as it has been said, these equations allow a prediction of the biological activity. When there is information collected from the literature or from a laboratory, a statistical tool called multiple linear regression is used, taking as a dependent variable the values of biological activity of ligands and as independent variables, the calculated descriptors.

The time of a molecular simulation carried out by means of computational tools is much less than the time it would take for the synthesis and bioassays of new compounds, which could be months or even years. This advantage allows to take a series of molecules and thanks to the speed of having the results, directly feed the synthesis laboratory in the continuous process of the project. Thus, QSAR predicts new structures never seen before and proposes them to the organic chemists to be taken to the bioassays whose results confirm or contradict the values predicted by the QSAR model. In an optimal case, through this operational cycle, better candidates are obtained than through pure trial and error. This saves time, money, resources and avoids failure for those who develop new drugs.

The advantages of QSAR are the low cost, since it does not use laboratory instruments, nor chemical reagents, and in addition, there is free software for the generation of the models that provide interfaces that facilitate the handling and design. In addition, the construction of the molecules and the calculation of descriptors can be extremely fast. Among its disadvantages we can mention the need for training in computational methodologies (different operating systems and graphic interfaces, database management, software development) and in this sense, the resolution of different computational problems (compatibility, updates, records, data formats) as well as the fact of having to have data on biological activity of the molecules coming from the same source, the change of perspective in the way of working, etc.

### 3.2 Molecular descriptors

Molecular descriptors (MD) play a key role in many areas of research. They can be defined as numerical representations of the molecule that quantitatively describe its physicochemical information. But not all the information contained in a molecule, but only a part, can be extracted through experimental measurements. In recent decades there has been an increasing focus on how to capture and convert, in a theoretical way, the information encoded in the molecular structure into one or more numbers that are used to establish quantitative relationships between structures and properties, biological activities and other experimental properties. In this way, MDs have become a very useful tool to carry out the search for similarities in molecular repositories, since they can find molecules with similar physicochemical properties according to their similarity to the values of the calculated descriptors.

From the beginning of its application, thousands of molecular descriptors have been defined, which encode molecules in different ways, being able to give a generic description of the whole molecule (1D descriptors), whose calculation is simpler than those descriptors that define properties calculated from two- and three-dimensional (2D and 3D) structures, which define more specific characteristics, but whose calculation is more complex.

It has been argued that the number of atomic and molecular descriptors developed to date constitute a sufficient arsenal for the search for new drugs to develop. However, one of the causes of the lack of adjustment in the models may be the very nature of the sample or the inappropriate selection of the structural descriptors. The later may be due to the selection procedure used, or to the insufficient capacity to describe the phenomenon by the models. All this is reason enough to continue the search for new structural or atomic descriptors that can be used in QSAR-based model studies.

The molecular descriptors can be divided into two main categories. Experimental measurements, such as log P, molar refractivity, dipole moment, polarisability and, in general, additive physical–chemical properties and theoretical molecular descriptors, which are derived from a symbolic representation of the molecule and can be further classified according to the different types of molecular representation. Theoretical ones, in turn, are classified into:

1. **Constitutional:** reflect general properties of molecular nature
2. **Topological:** its calculation is done through graph theory
3. **Geometric:** are derived from empirical schemes and encode the ability of the molecule to participate in different types of interactions.
4. **Electronics:** refer to the electronic properties
5. **Physicochemicals:** define the behaviour of the molecule in the face of external reactions

If we consider the dimensions of the molecular characteristics represented by the theoretical molecular descriptors, the following categories are established.

#### 3.2.1 0D descriptors

Are the easiest to calculate and interpret. Included in this category are all those molecular descriptors for whose calculation no structural information of the molecule or connectivity between atoms is needed and therefore they are independent of any conformation problem and do not need optimization of the molecular structure.

They usually show a very high degeneration, that is, they have equal values for several molecules, such as isomers. Their information content is low, but they can nevertheless play an important role in the modelling of various physicochemical properties or participate in more complex models. Examples of these descriptors are the number of atoms, number of bonds of a certain type, molecular weight, average atomic weight or sum of atomic properties such as Van der Waals volumes.

#### 3.2.2 1D descriptors

All the molecular descriptors that allow calculating information from fractions of a molecule can be included in this category. They are usually represented as fingerprints, which are no more than binary vectors in which 1 indicates the existence of a substructure and 0 indicates its absence. This form of representation has a great advantage and is that it allows calculations to be carried out very quickly to find similarities between molecules. Like 0D, these descriptors can be easily calculated, are naturally interpreted, do not require optimization of the molecular structure and are independent of any conformation problem. They usually show a medium–high degeneration and are often very useful to model both physicochemical and biological properties.

Within the 1D descriptors we talk about those based on the count of chemical functional groups, such as the total number of primary carbon atoms, number of cyanates, number of nitriles, etc, and the so-called atom-centred fragments, which are based on the count of different fragments of the molecule. Examples of the last-mentioned are hydrogen bonded to a heteroatom, hydrogen bonded to an alpha carbon and fluorine bonded to a primary carbon.

#### 3.2.3 2D descriptors

They describe properties that can be calculated from two-dimensional representations of molecules. They are obtained through the graph theory, independent of the conformation of the molecule. Their calculation is based on a graphic representation of the molecule and they present theoretical properties of structure that are preserved by isomorphism, i.e. properties with identical values for isomorphic graphs. The invariant part can be a characteristic polynomial, a sequence of numbers or a single numerical index obtained by applying algebraic operators to matrices representing molecular structures and whose values are independent of the numbering or labelling of the vertices.

They are generally derived from a molecular structure degraded in hydrogen. They can be sensitive to one or more characteristic structures of the molecule such as size, shape, symmetry, branching and cyclicity and can also encode chemical information about the type of atom and the multiplicity of bonds. In fact, they are generally divided into two categories:

1. **Structural-Topology index::** encode only information about the adjacency and distance of atoms in the molecular structure.
2. **Topochemical index:** quantify information on topology but also on specific properties of atoms such as their chemical identity or state of hybridization.

#### 3.2.4 3D descriptors

The three-dimensional descriptors are related to the 3D representation of the molecule and include the conformation of the molecular structure, where the distances between bonds, bond angles, dihedral angles, etc. are considered, being able then to describe the stereochemical properties of the molecules. Its calculation is more complex than in the previous ones and may require the analysis of many molecular conformations.

The most popular 3D descriptors include representations of pharmacophore type molecules, defined as a set of steric and electronic features needed to ensure optimal supramolecular interactions with a specific biological target and trigger or block its biological response, where features such as hydrophobic centres or hydrogen bond donors, which are known or believed to be responsible for biological activity, are mapped into positions in a molecule. The conformation-dependent distances between these points are then calculated and recorded. Three-point pharmacophores are widely used, but more potent four-point pharmacophores have been introduced, which may require the analysis of millions of possible pharmacophores for a test compound. Complex 3D descriptors are calculated, for example, to identify active conformations of a compound or to identify critical characteristics for differences in activity in series of analogues. At the same time, this type of calculation is needed to generate the “pharmacophore shape” of a query molecule in order to search databases for compounds with similar 3D characteristics. In addition, the use of pharmacophore type descriptors is fundamental for the derivation of 3D-QSAR or 4D-QSAR models.

#### 3.2.5 4D descriptors

Also referred to as grid-based, these descriptors provide additional information by introducing a fourth dimension that allows characterization of interactions between molecules, their conformational states and the active sites of a biological receptor. The central hypothesis is that consideration of ligand conformational variation, influenced by factors such as solvent molecules and non-covalent interactions within protein binding pockets, will result in descriptors that characterize the molecular properties of compounds more accurately and thus lead to more reliable QSAR models.

From the work published by Cramer et al. in 1988 [22], the use of the field properties of the molecules was proposed field properties of molecules in three-dimensional space to develop and apply QSAR models. This method was called Comparative Molecular Field Analysis (CoMFA) and its basic foundation consists of sampling the steric (van der Waals) and electrostatic (Coulombic) fields around a set of aligned molecules, in order to capture all the information necessary to explain the final response exhibited by the molecules. The sampling consists of calculating the interaction energies of the molecules, by means of appropriate probes located in the three-dimensional lattice arranged around the molecular structures.

A critical step in the CoMFA method is the proper alignment of the molecules, which can be time-consuming and requires prior knowledge of the precise molecular conformation. For this reason, it is necessary to analyze the most active molecules in the dataset and use them as a template. Additionally, when the dataset features molecules with several conformational degrees of freedom, the selection of the active conformation is often a significant hurdle in QSAR-3D modeling. Therefore, a prior systematic conformational search must be performed to define the most stable conformer according to the generated Potential Energy Surface (PES). Currently, there are different computational methods to improve the performance of the CoSAR-3D alignment performance of the CoMFA technique [23].

Most QSAR models use numerical descriptors derived from the two- and/or three-dimensional structures of molecules. Conformation-dependent characteristics of flexible molecules and interactions with biological targets are not encoded by these descriptors, leading to limited prediction. 2D/3D QSAR models are successful for virtual screening, but often suffer in the optimization stages. That’s why conformation-dependent 4D-QSAR modeling was developed two decades ago, but these methods have always suffered from the associated computational cost. Recently, 4D-QSAR has undergone a significant breakthrough due to rapid advances in GPU-accelerated molecular dynamics simulations and modern machine learning techniques [24].

During the last 15 years, new multidimensional descriptors have been incorporated into QSAR modeling, termed 5D and 6D descriptors. These are based on structural parameters associated with the flexibility of the receptor binding site along with the topology of the ligand. Specifically, the 5D descriptors are calculated from multiple conformations, orientations, protonation states and stereoisomers of the ligand under analysis. In the case of the 6D descriptors, it is necessary to take into account the solvation scenarios of the complex, the ligands and the interacting environment[25].

#### 3.2.6 How many descriptors are needed?

In broad terms, the number of descriptors to be used will depend on the computational tools available and the number of molecules included in the study. The most frequent error consists of allowing the mathematical operation of the linear regression model to add an over-dimensional numerical space to describe each molecule independently without being able to establish a rule with predictive and reliable power. This happens when the number of descriptors exceeds the number of molecules. On the other hand, the exhaustive search, can be applied to all but the simplest cases, since the search space is not practical when there is a low number of molecular descriptors. The reliability of the model can be affected, not only by the presence of noise, but also by the correlation of redundant descriptors and also by the presence of irrelevant descriptors. Therefore, variable selection techniques are largely used to remedy this situation and improve the accuracy and predictive power of classification or regression models [16].

In recent years, the scientific community has focused much attention on techniques dedicated to variable selection, i.e. the selection of molecular descriptors in QSAR. Since there are thousands of descriptors available to describe a molecule and often there is no a priori knowledge about which characteristics are most responsible for a specific property, subsets of the most appropriate descriptors are explored through different strategies. Today there are many software tools for calculating molecular descriptors, each with its advantages and disadvantages (ease of use, licences, number of descriptors, etc.).

### 3.3 Fingerprints

Fingerprints (FP) are a particular form of molecular descriptors that allow quickly and easily the effective representation of the molecular structure through a chain or vector of bits, with a fixed length, which indicate the existence or absence of internal substructures or functional groups. This form of molecular coding is very efficient for storing, processing and comparing the data that is hosted in the strings that contain the molecular information. However, fingerprints that are derived from chemical structures ignore the biological context, thus leaving a gap between molecular structure and biological activity, so that small changes in the former can produce substantial differences in bioactivity.

**Fig. 4:**
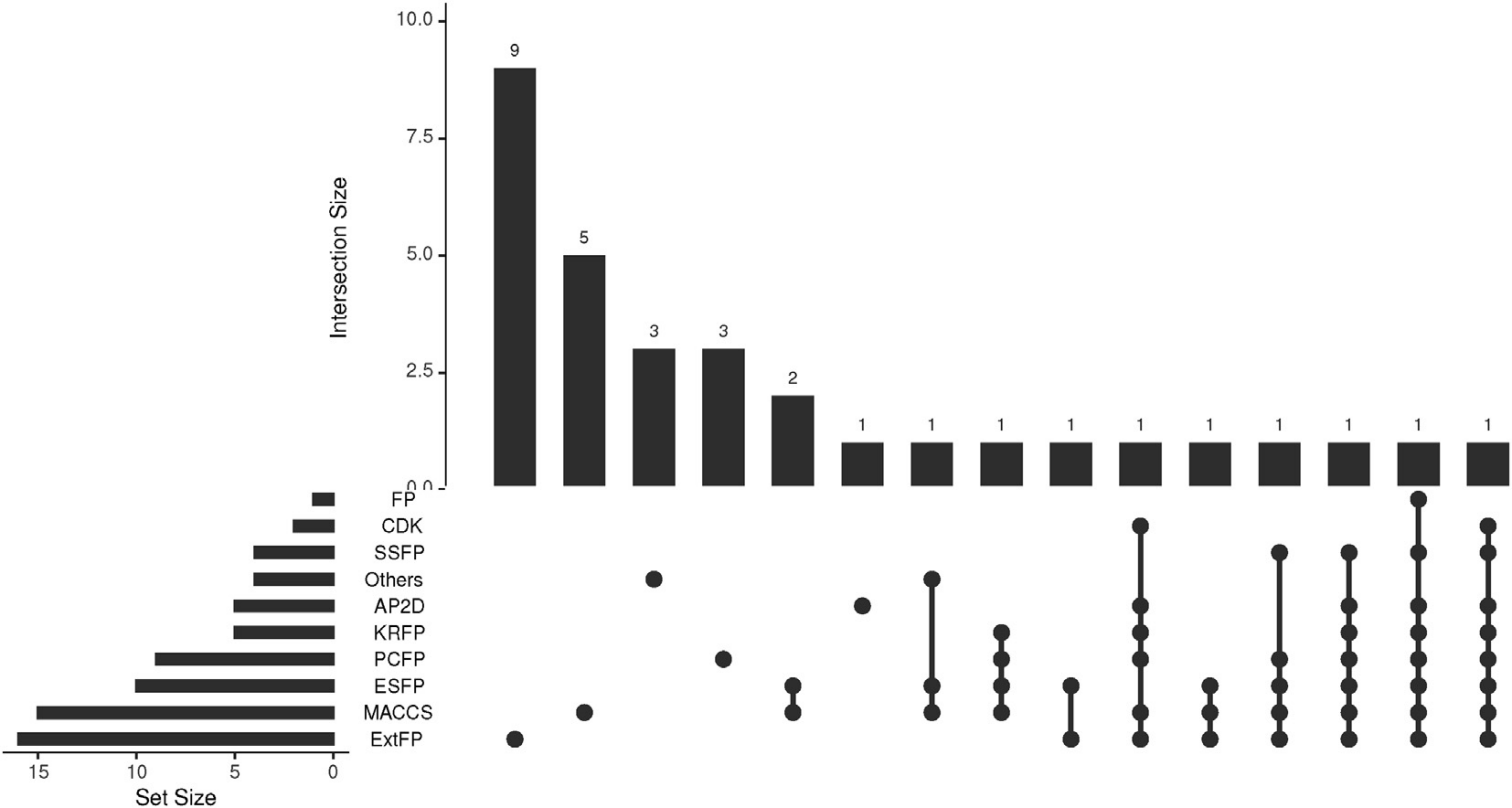
The number of identified items that have used the most common fingerprints is represented.

There is a wide variety of FP, from the simplest, which lists a catalog of 2D substructures (e.g. MACCS), to more advanced versions that include 3D information on molecular conformation. The following is a summary of the most commonly used ones. In the Fig. 4 a summary of the descriptors found in the articles consulted is shown. It indicates the number of times a descriptor appears individually in a publication, as well as the number of studies that have used several of them. It is common in this type of study to compare several of them. The Fig. 4 shows how ExtFP fingerprints are the most used in absolute terms. In second place we find the MACCS. The main reasons for their extensive use are their easy calculation and the positive results they have been obtaining in the different problems. Bearing in mind that the sample of articles consulted is representative of the last five years of research, we can see how these descriptors are still used today in research, since their use has not been diminished by the appearance of other more sophisticated analysis techniques.

#### 3.3.1 Extended-Connectivity Fingerprints

Extended Connectivity Fingerprints (ECFP) are a class of topological fingerprints for molecular characterization [26], [27]. Historically, topological fingerprints were developed for the search of substructures and similarities, but these have been developed specifically for structure–activity modeling [28], [29], [30], [31], [32].

ECFPs are circular fingerprints with a number of useful qualities:

1. They can be calculated very quickly.
2. They are not predefined and can represent a large number of different molecular characteristics (including stereochemical information).
3. Its characteristics represent the presence of particular substructures, which allows an easier interpretation of the analysis results.
4. They are designed to represent both the presence and absence of functionality, as both are crucial for analyzing molecular activity.
5. The ECFP algorithm can be adapted to generate different types of circular fingerprints, optimized for different uses.

They are among the most popular similarity search tools in drug discovery [33], [34], [35], [36], [37], [38] and are used effectively in a wide variety of applications. They can store information about the environments surrounding each atom in a molecule and in addition to the search for similarities, ECFPs are well suited to recognizing the presence or absence of particular substructures, so they are often used in the construction of QSAR and QSPR models.

#### 3.3.2 MACCS keys

MACCS (Molecular ACCess System) Keys are another of the most used structural keys [36], [37], [39], [38]. Sometimes they are known as MDL keys, which bear the name of the company that developed them. While there are two sets of MACCS keys, one with 960 keys and the other containing a subset of 166 keys [40], [41], [42], [43], [44], only the shortest fragment definitions are available to the general public. These 166 public keys are implemented in open source chemoinformatics software packages, including RDKit, CDK, etc.

#### 3.3.3 Pubchem fingerprints

PubChem is a repository that houses a large amount of molecular information that can be consulted and downloaded for free. A substructure is a fragment of chemical structure for which PubChem generates a fingerprint, which is a list of bits [41], [42], [45]. PubChem Fingerprints are 881-bit long structural keys, which PubChem uses to perform similarity search [44], [46]. It is also used for neighboring structures, which pre-calculate a list of similar chemical structures for each compound. This pre-calculated list can be accessed through the Compound Summary page.

#### 3.3.4 Atom pairs

They are fingerprints based on topological routes, which represent all possible connectivity routes defined by a specific fingerprint through an input compound [36], [39], [37], [41]. They mainly focus on chemical connectivity information of synthetic compounds. In turn, within this classification we can distinguish:

1. AtomPairs2DFingerprint (APFP): they are defined in terms of the atomic environment and the shortest path separations between all pairs of atoms in the topological representation of a composite structure. It encodes 780 pairs of atoms at various topological distances.
2. GraphOnlyFingerprint (GraphFP): is a specialized version of the molecular fingerprint in the Chemical Development Kit (CDK), which encodes the 1024 path of a fragment in the composite structure and does not take into account the information of the binding order.

#### 3.3.5 CDK

The Chemical Development Kit (CDK) is a set of widely used open source chemoinformatics tools (drug discovery, toxicology, etc), which provides data structures to represent chemical concepts, including 2D and 3D representation of chemical structures, along with methods to manipulate these structures and perform calculations on them. The library implements a wide variety of algorithms ranging from the canonicalization of chemical structure to molecular descriptor calculations and the perception of pharmacophores [36].

The CDK provides methods for common tasks in molecular computing, including 2D and 3D representation of chemical structures, SMILES generation, ring searches, isomorphism verification, structure diagram generation, etc. Implemented in Java, it is used both for server-side computational services, possibly equipped with a web interface, and for client-side applications [37].

#### 3.3.6 Other types of fingerprints

In addition to the types of fingerprints described above, there are many other types that, although not as widely used, do appear in the literature very often, such as EstateFP [42], [47], [48] and Klekota-Roth [39], [41], [36].

### 3.4 Graph-based machine learning algorithms

Most predictive models in chemoinformatics base their input data on molecular descriptors calculated and coded in numerical vectors, as described in the previous section. The use of these descriptors generates high dimensionality matrices for the use of classical ML algorithms such as Random Forest, SVM, ANN, NB, etc. These algorithms are designed to process data structured in matrices or vectors, but are not capable of using the total information of molecules that are represented as a mathematical graph.

A molecular network is the representation of the structural formula of a chemical compound in terms of graph theory. In terms of the representation of a molecule each molecule is represented as a graph (G). Each atom in the network is represented as a node in the network. V is the set of atoms in the molecule, A corresponds to the adjacent matrix that indicates the connectivity between atoms, and the X matrix represents the atomic characteristics for each atom. Therefore, each graph is mathematically represented as G = (V, A, X) [49], [50].

Recently the creation of chemoinformatic models, capable of predicting specific functions, were based on the information extracted from these molecular graphs. For this purpose, the algorithms used were artificial neuron networks. Unlike more conventional topologies such as the fully connected neural network (FNN) or the convolutional neural network (CNN), which extract information from vectors or numerical matrices, graph neural networks (GNNs) are capable of extracting structural information from a mathematical graph. In January 2009 when Scarselli et al. [51] presented The Graph Neural Network Model, and from that moment these models were widely used in various applications. Among them, chemoinformatics was the one that has grown significantly in the last decade.

Subsequently, several works were published for the improvement and applicability of these graph-based models. For example, Duvenaud et al. [52] presented an architecture based on the generalization of fingerprint computing so that it can be learned through retropropagation. On the other hand, Bruna et al. [53] introduced convolutional deep networks on spectral representations of graphs, while Masci et al. [54] described the convolutional networks on non-Euclidean collectors. Graphics-based machine learning algorithms, in particular GNN, have recently begun to attract significant attention in chemical science [55], [56], [57], [51].

Graph convolutions are a deep learning architecture for learning directly from undirected graphs. In 2016, researchers from Stanford and Google Inc. developed what is known as Molecular Graph Convolutions [58]. It was as a result of the application of convolutional algorithms for graphs that computational research in drug discovery has taken a step forward. In the last years many researches have been published using this kind of algorithms or variants for a certain function [49]. For example, at work [59] propose a robust and guided molecular representation based on Deep Metric Learning, which automatically generates an optimal representation for a given data set. In this way they try to solve the modifications generated in the properties of the molecules to changes in its molecular structure. On the other hand, in [60] developed new network definitions using the assigment of atom and bond types in the force fields of molecular dynamics methods as node and edge labels, respectively. Thus improving the accuracy of classification activities for chemicals. In addition, these algorithms can be combined with others such as [61]. In this work they designed new drugs based on GCN and learning by reinforcement. In this case, they addressed the problem of generating new molecules with desired interaction properties as a multi-target optimization problem. They use trained GCN with linkage interaction data. The combinations of these terms, including drug similarity and synthetic accessibility, were optimized using a reinforcement learning based on a graphical convolution policy approach. Moreover, in [62] propose a comprehensive method to apply symmetry in the graphical neural network, which extends the coverage of the prediction property to the orbital symmetry for both normal and excited states. This method is able to include the molecular symmetry in the predictive models linking the real space (R) and the moment space (K). Finally, another example is the one performed by [63], where they implemented graph-based deep learning models to predict flash points of organic molecules, which play an important role in preventing flammability risks. After comparing them with different techniques, they observed how the graph-based models outperformed the others. On the other hand, in [55] implemented another graph-convolutional neural network for the prediction of chemical reactivity.

One of the weaknesses of these technologies is that most of the times they are treated as black boxes, which present a very low interpretability of the results. To avoid this fact, in [64] develop a new graphical neural network architecture for molecular representation that uses a graphical attention mechanism to learn from relevant drug discovery data sets. The interpretability of the results obtained by this new architecture stands out from this work.

## 4 Biological problems asses by Machine Learning in drug discovery

A drug can be defined as a molecule that interacts with a functional entity in the organism, called a therapeutic target or molecular target, modifying its behaviour in some way. Known drugs act on known targets, but the discovery of new ones that can modify the course of a disease or improve the effectiveness of existing treatments is one of the main objectives of research in the field of chemistry and biology.

The development of a new drug can take up to 12 years and it is estimated that its average cost, until it reaches the market, is approximately one billion euros. The time and costs involved are largely associated with the large number of molecules that fail at one or more stages of their development, as it is estimated that only 1 in 5,000 drugs finally reach the market.

The previous statistics show that the discovery and development of new drugs is a very complex and expensive process. This process has been carried out for a long time using exclusively experimental methods. The technological advances of the last few decades have promoted the birth of the term *in silico*, a term that is now common in biology laboratories, and which designates a type of experiment that is not done directly on a living organism (these are called *in vivo* experiments) or in a test tube or other artificial environment outside the organism (experiments called *in vitro*), but is carried out virtually through computer simulations of biological processes.

The complexity of modern biology has made these computational tools essential for biological experimentation, as they allow theoretical models to be coded with great precision and are capable of processing large amounts of information, thus facilitating and accelerating the process of developing new drugs.

**Fig. 5:**
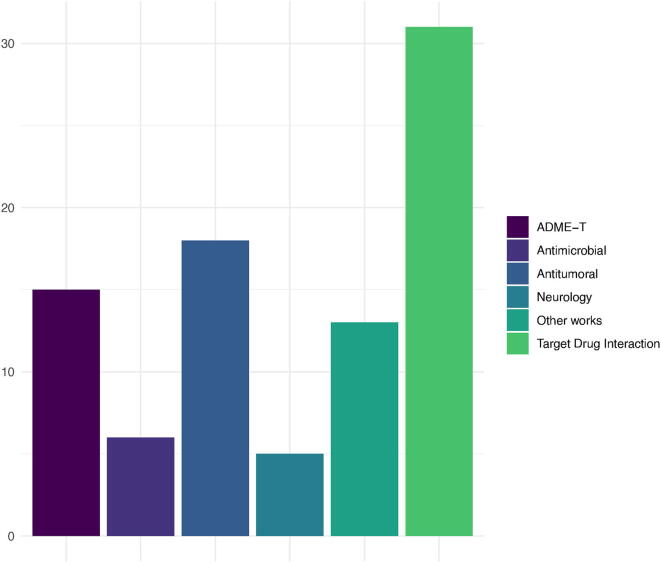
Counting of identified articles classified according to the biological problem addressed. The sampling of selected articles was during the period from 2016–2020.

The development of a new drug begins with the search phase, through high-performance screening, for so-called *hits*, a term used to describe those molecules or compounds that show biological activity against a therapeutic target or molecular target, which is the place in the body where the drug is intended to operate. This phase is followed by the generation of *leads*, where the previously selected molecules are validated and structurally refined to increase their potency with respect to the target. In addition, appropriate pharmacokinetic properties are expected, i.e. adequate absorption, distribution, metabolism and elimination (ADME) rates, as well as low toxicity and adverse effect rates. A bar chart that quantifies the number of items identified in various biological problems is shown in Fig. 5. These problems have been defined by the researchers according to the set of articles consulted. It can be seen that the vast majority of the articles belong to the group of therapeutic targets. On the other hand, it can be seen that the works related to cancer are of great interest, as well as those aimed at predicting the adverse effects of certain drugs.

### 4.1 Administration, distribution, metabolism, elimination and toxicity

The concept of drug similarity, established from the analysis of the physicochemical properties and structural characteristics of existing or candidate compounds, has been widely used to filter out compounds with undesirable properties in terms of administration, distribution, metabolism, elimination and toxicity (ADMET) [65]. The study of the ADME phases that a drug undergoes after being administered to an individual is another of the fundamental tasks in the development of new compounds [66]. Alteration in a patient of any of these stages (for example, excretion problems due to some type of renal failure, increased volume of distribution in obese people, absorption problems due to gastrointestinal pathology or problems in the metabolism of the drug due to deterioration of liver function) may influence the final plasma concentration of the drug modifying the expected response of the organism, thus requiring a decrease or increase in the dose of the drug in each case.Therefore, it is essential in the early stages of research to estimate the behaviour of the pharmacokinetic properties of a compound, and new tools have been developed to improve and speed up this phase of development. This is the example of Chemi-Net [67], for the prediction of ADME properties, which increases the accuracy over another tool with the same purpose already in existence.

The company Bayer Pharma has implemented a platform for absorption, distribution, metabolism and excretion ADMET *in silico* with the aim of generating models for a wide variety of useful pharmacokinetic and physicochemical properties in the early stages of drug discovery, but these tools are accessible to all scientists within the company [68].

The octanol–water partition coefficient is a measure of the hydrophobicity or lipid affinity of a substance dissolved in water. Chemical compounds with high values of this coefficient usually accumulate in the lipid portions of the organisms, thus producing toxicity. On the contrary, the compounds with low coefficient tend to be distributed in water or air, so they could be eliminated from the organism without accumulating [69]. Furthermore, the adequate estimation of the half-life of elimination of a drug would have potential applications for the first pharmacokinetic evaluations and thus provide guidance for designing drug candidates with a favourable *in vivo* exposure profile [70], so improving this estimation is another grain of sand in the development of the complex process.

The metabolism is the main route of elimination from the body for most of the 200 most marketed medicines. The study of the stability of NADPH-fortified liver microsomes is common in the research of new drugs to predict clearance and thus be able to estimate the maximum exposure to a drug for a given dose [35]. In addition, the liver is the main organ involved in drug metabolism, and therefore liver injury caused by drugs has often hindered the development of new drugs. Assessment of the risk of liver injury to drug candidates is an effective strategy to reduce the risk that a study will not go ahead with new drug discovery.

Toxicity is a major cause of the failure of drug research and development. International data showed that in the period from 2006 to 2010, toxicity accounted for 22 and 54% of failures in drug research and development, at the clinical and preclinical stages, respectively. Adverse drug reactions (ADRs), which can increase morbidity, occur more significantly in hospitalized patients and, in addition to the clinical burden, also represent a significant economic cost. For all these reasons, early stage virtual screening of drug candidate molecules plays a key role in the pharmaceutical industry to prevent ADR. It is therefore essential to study toxicological properties as early as possible and to give priority to the main compounds that pose the least threat at the stage of discovery of hits, thus increasing the chances of success during clinical development.

Computer-based predictions of toxicity and ADR can point drug safety testing in the right direction and consequently shorten the time required and save costs during drug development. Some adverse reactions can be part of the natural pharmacological action of a drug that cannot be avoided, but more often, they can be unpredictable at the development stage. They may occur due to the administration of one drug or the combined use of two or more drugs. The aim of [71] is to identify treatments for dermatological diseases (including psoriasis, atopic dermatitis, rosacea, acne vulgaris, alopecia, melanoma, eczema, keratosis, and pruritus) that may induce adverse reactions when taken in combination with other topical or oral drugs (immunosuppressants, enzyme inhibitors) prescribed to treat other pathologies (fungal/bacterial infections).

To advance research into compounds against infectious agents, these compounds must show a relative lack of toxicity in mammalian cells. In initial trials, the use of Vero cells to measure the cytotoxicity that a drug can produce is common, thus allowing the identification of non-cytotoxic components [28].

Prior to clinical application of a drug, it must go through two stages of ADR detection, including pre-clinical, to study safety profiles and clinical safety trials of drugs [72]. The high number of potential adverse effects that can occur with the consumption of drugs alone or in combination, makes it difficult to detect many of these adverse effects during early drug development, so tools such as SDHINE [73] have been proposed to predict adverse drug reactions. In [74], they are developing a new methodology for predicting drug side effects (’Feature Selection-based Multi-Label-K-Nearest Neighbor method’), which can also help reveal the possible causes of adverse effects. In general, computational tools have also been developed to distinguish between carcinogenic and non-carcinogenic compounds [37].

Many drug candidates can cause blockage of potassium channels, a potentially fatal phenomenon as it can produce a long QT syndrome, leading to death from ventricular fibrillation. In their work [67] they provide a web-based tool for predicting cardiotoxicity of potassium channel-related chemicals hERG [75], which can be used in the high-performance virtual detection stage for drug candidates.

Computational attempts have also been made to evaluate toxicity profiles of compounds included in drugs used in the treatment of HIV and Malaria [76].

### 4.2 Antimicrobials

Infectious diseases are caused by pathogenic microorganisms such as bacteria, viruses, parasites or fungi. These diseases can be transmitted, directly or indirectly, from one person to another.

Few new classes of antibiotics have emerged in recent decades and this pace of discovery it is unable to keep up with the increasing prevalence of resistance. However the large amount of data available promotes the use of machine learning techniques in discovery projects (for example, building regression, classification models and virtual classification or selection of compounds). The authors of [77] review some Machine Learning applications focusing on the development of new antibiotics, the prediction of resistance and its mechanisms.

Antibiotics are the treatment of choice for bacterial diseases, but the increase and abuse of antibiotics has led to the emergence of bacterial resistance to many of today’s antibiotics, hence the need to generate new compounds that can combat multi-resistant organisms. Thus, much research is being done on Halicin [78], which is a molecule with bactericidal capacity that has shown great promise in attacking bacteria that are difficult to treat with current antibiotics. Its structure is different from that of conventional antibiotics and shows bactericidal capacity against a wide phylogenetic spectrum that includes Mycobacterium Tuberculosis (bacteria for which more efficient treatments are still being sought [31]), and enterobacteria, clostridium difficilae and acinetobacter baumanii. But resistance is not exclusive to bacteria, and resistance to current treatments for malaria, produced by the protozoan parasite plasmodium falciparum, is a concern, affecting more than 200 million people worldwide. While the treatment of choice includes combinations of drugs [79], studies are already underway to identify new combinations that may circumvent current resistance mechanisms [80].

Viruses can cause common infectious diseases such as the common cold and influenza, for which there is currently no specific treatment (it is the immune system that eliminates it from the body) or preventive treatment (vaccines). But the viruses also cause serious diseases such as AIDS, Ebola (which is the cause of a large number of studies [81] because of the 2016 epidemic), or COVID-19 (on whose curative or preventive treatment most current global efforts are focused). In addition, co-infection with certain viruses is common in certain populations, such as co-infection with human immunodeficiency virus type 1 and hepatitis C virus (HIV/HCV). In this case treatment of coinfection is a challenge due to the special considerations to be taken into account to ensure liver safety and avoid drug interactions. Therefore, drugs that are effective against multiple pathogens and with less toxicity that can provide a therapeutic strategy in certain co-infections are sought [38].

An effective therapeutic strategy is urgently needed to treat the rapidly growing COVID-19 in patients from all over the world. As there is no proven effective drug to treat COVID-19 patients, it is fundamental to develop efficient strategies that allow the reuse of drugs or the design of new drugs against SARS-CoV-2.

In [82] the main strategies are summarized that are being used through the use of artificial intelligence and machine learning techniques.

The 3Clike protease of the coronavirus, associated with the syndrome severe acute respiratory tract (or 3CLpro), is a potential target as anti-SARS agents, due to its critical role in viral replication and transcription. Due to the high structural closeness between the enzymes in the old strain SARS CoV and the new SARS CoV-2, it would be expected that compounds that inhibit the former enzyme would show similar interactions with the latter. In numerous studies [83], [84], QSAR models have been developed to search for compounds that act as SARS-CoV-3CLpro enzyme inhibitors and a study of the structural characteristics of those molecules that control its inhibition of 3CLpro (pIC50).

Central nervous system (CNS) infections are a major cause of morbidity and mortality. The passage of fluids and solutes into the CNS is closely regulated through the blood–brain barrier (BBB). The penetration of any drug into the cerebrospinal fluid (CSF) depends on molecular size, lipophilicity, binding to plasma proteins and their affinity for transporters. In the search for drugs indicated for CNS infections, it is essential to predict their capacity to cross this barrier [85], being able to discard from the beginning those that do not do so in minimal concentrations.

### 4.3 Target drug interaction

Determining the goal to be reached is the most important, and most error-prone, in the development of a therapeutic treatment for a disease, where failures are potentially costly given the long time frames and expenses of drug development. Compound-protein interaction (CPI) analysis has become a crucial prerequisite for new drug discovery [86], [87], [88], [89], [90]. *In vitro* experiments are commonly used to identify CPIs, but it is not feasible to perform this task through experimental approaches alone, and advances in machine learning in predicting CPIs have made great contributions to drug discovery. To improve the task of predicting ligand–protein interactions, tools such as Multi-channel PINN [26]. In addition, the study of these interactions is essential for obtaining traces of novel drugs and predicting their side effects from approved drugs and candidates [40].

On the other hand, the identification of the viability of the target proteins is another of the preliminary steps of drug discovery [91], [92], [93]. Determining a protein’s ability to bind drugs in order to modulate its function, called drug capacity, requires a non-trivial amount of time and resources. This task is aided by new functions developed in eFindSite [94], which is independent software available free of charge, that uses supervised machine learning to predict the pharmacological ability of a given protein.

The crystallised ligands in the Protein Data Bank (PDB) can be treated as inverse forms of the active sites of the corresponding proteins. The similarity of shape between a molecule and the ligands of PDBs indicates the possibility of the molecule binding to certain targets [95]. Membrane proteins are involved in many essential biomolecule mechanisms as a key factor in enabling the transport of small molecules and signals on both sides of the cell membrane. Therefore, accurate identification of membrane ligand–protein binding sites will significantly improve drug discovery. To this end, MPLs-Pred [96] has been developed as a freely available tool for general users. The prediction of interactions from Multi Kernel methods makes it possible to identify possible drug-target interaction pairs [97].

Cone snails are poisonous sea snails that inject their prey with a lethal cocktail of conotoxins, small, secreted, cysteine-rich peptides. Given the diversity and often high affinity for their molecular targets, which consist of ion channels, receptors or transporters, many conotoxins have become valuable pharmacological probes. However, homology-based search techniques, by definition, can only detect new toxins that are homologous to previously reported conotoxins. To overcome these obstacles ConusPipe is an automatic learning tool that uses chemical characters of conotoxins to predict whether a certain transcript in a Conus transcriptome, which has no otherwise detectable homologues in current reference databases, is a putative conotoxin. This provides a new computational pathway for the discovery of new families of toxins [98].

### 4.4 Antitumorals

Cancer is a major public health problem throughout the world and it is therefore imperative that new drugs are developed for its treatment. The main goal of cancer research is to discover the most effective method of treatment for each cancer patient, since not everyone responds equally to a specific treatment due to external factors, such as the use of tobacco products and unhealthy diets, and internal factors, such as heterogeneity of cancer cells and immune conditions. As the number of cancer patients worldwide increases each year, being able to correctly predict a cancer’s response or non-response to a specific drug would be invaluable.

The word cancer refers to the uncontrolled proliferation of abnormal cells, which when they outgrow normal cells, make it difficult for the body to function the way it should. Although the word cancer is used in a general way, the term encompasses a range of diseases with very different pathogenesis, evolution and treatment.

In general, many therapeutic targets have been studied and identified that may be amenable to treatment. Computational techniques have been widely used to predict the activity of many compounds on these targets, so for example it is known that G-protein-coupled receptors (GPCRs) play a key role in many cell-signalling mechanisms whose alteration may be involved in the pathogenesis of cancer [99]. Protein 4, which contains bromodomain (BRD4), has emerged as a promising therapeutic target for many diseases, including cancer, heart failure and inflammatory processes. Nitroxoline is an antibiotic that showed potential over BRD4 with inhibitory activity against leukaemia cell lines, and has been shown to be effective against leukaemia cell lines [100]. Indolamine 2,3-dioxygenase (IDO), an immune checkpoint, is a promising target for cancer immunotherapy. Three IDO inhibitors with potent activity have been identified by machine learning methods [34], but have not yet been approved for clinical use. Attempts have also been made to predict the response to the same drug of different types of tumours [101], including breast cancer, triple-negative breast cancer and multiple myeloma. Computational methods have also been used to study histone deacetylases (HDAC), which are an important class of enzyme targets for cancer therapy, and inhibitory compounds of these have been sought through computational techniques [102].

The phosphoinositide 3-kinase protein (PI3K) plays a key role in an intracellular signalling pathway responsible for many processes in response to extracellular signals, such as regulation of the cell cycle, cell survival, cell growth, angiogenesis, etc. Vascular tumours in children often show mutations in this molecule, so it has become a promising drug target for cancer chemotherapy. PI3K inhibitors have gained importance as a viable cancer treatment strategy as they control most features of cancer, including cell cycle, survival, metabolism, motility and genomic instability. Structure-based virtual screening has been performed to identify PI3K inhibitors [103]. Another membrane glycoprotein studied as a target, and for which inhibition simulations have been carried out, is P-gp membrane laglucoprotein [104]. Different tumour cell lines have also been used to study antibodies as a possible treatment [105] quantifying levels of proliferation and apoptosis to predict their functioning.

Molecules can be identified as anti-cancer agents through two widely used drug discovery methods [106]: target-based drug discovery (TDD, target-first, direct chemical biology) and phenotype-based drug discovery (PDD, function-first, reverse chemical biology).

Kinases are one of the largest families considered to be attractive pharmacological targets for neoplastic diseases due to their fundamental role in signal transduction and regulation of most cellular activities [107]. As a result, kinase inhibitors have gained great importance in cancer drug discovery over the last two decades, because despite considerable academic and industry effort, current chemical knowledge of kinase inhibitors is limited and therefore tools such as Kinformation [108] have been developed, which is based on machine learning methods to automate the classification of kinase structures and this approach is expected to improve protein kinase modelling in both active and inactive conformations.

Neoplastic growth and cell differentiation are fundamental characteristics of tumour development. It is well established that communication between tumour cells and normal cells, through channels containing connective tissue including gap bonds, tunneled nanotubes and hemochannels, regulates tumour differentiation and proliferation, aggressiveness and resistance to treatment. It has been proposed that new computational approaches [109] to the identification and characterisation of these communication systems, and their associated signalling, could provide new targets for preventing or reducing the consequences of cancer.

It has been postulated that new drug combinations can improve personalized cancer therapy. Using various types of genomic information on cancer cell lines, drug targets and pharmacological information, it is possible to predict drug combination synergy by regressing the level of synergy between two drugs and a cell line, as well as classifying whether synergy or antagonism exists between them [110].

For the current diagnosis of many cancers, nuclear morphometric measurements are used to make an accurate prognosis in the last stage, but early diagnosis remains a great challenge. Recent evidence highlights the importance of alterations in the mechanical properties of individual cells and their nuclei as critical drivers for the emergence of cancer. Detecting subtle changes in nuclear morphometry at single cell resolution by combining fluorescence imaging and deep learning [111] allows discrimination between normal cells and breast cancer cell lines, thus opening new avenues for early disease diagnosis and drug discovery.

### 4.5 Neurology

Chemotherapy-induced peripheral neuropathy (CIPN) is a common adverse side effect of cancer chemotherapy, which can cause extreme pain and even disable the patient. Lack of knowledge about the mechanisms of multifactorial toxicity of certain compounds has prevented the identification of new treatment strategies, but computational models of drug neurotoxicity [112] are used early in drug development to detect high-risk compounds and select safer candidate drugs.

Many CNS disorders, both neurodegenerative processes and trauma, require multiple strategies to address neuroprotection, repair and regeneration of cells. The knowledge accumulated in neurodegenerative processes and neuroprotective treatments can be used, through computational techniques such as Machine Learning, to identify combinations of drugs that can be reused as potential neuroprotective agents [113]. Another branch of neurology that generates great scientific interest is the study of neurodegenerative diseases, such as Alzheimer’s disease, the main cause of dementia and pathology that currently has no cure. Several studies have reported that the expression of ROCK2, but not of ROCK1, has increased significantly in the human nervous tissue of patients with neurodegenerative disorders, so that the suppression of the expression of ROCK2 is considered a pharmacological target for the treatment of this disease [48]. In the same sense, 5-HT1A is a brain receptor used as a biomarker for degenerative disorders. Work has been carried out to predict compounds that will bind to this receptor [114]. In general, based on an equal number of drugs approved or withdrawn for the treatment of CNS pathologies, possible discriminative fragments have been studied that allow the search for other similar compounds for the treatment of CNS pathologies [115].

### 4.6 Other works

Type 2 diabetes mellitus is the most common endocrine pathology in the world, causing many complications in many organ systems that can lead to a shortening of life and a considerable reduction in the quality of life of patients suffering from it. For this reason, the pharmaceutical industry has made many efforts in the search for efficient treatments that can cure this disease or, failing that, minimize the lesions produced in target organs by excess blood glucose. One of the branches of research focuses on the inhibition of sodium-dependent glucose co-transporters (SGLT1 and SGLT2), through which glucose is absorbed. Dual inhibitors have been developed, but the search continues for compounds aimed at reducing the absorption of glucose by SGLT1 [33]. Another branch of research within endocrinology focuses on the physiopathology and treatment of obesity. It is known that nuclear receptors PPARs (Peroxisome Proliferator Activated Receptors) are transcription factors that are activated by the binding of specific ligands and regulate the expression of genes involved in lipid and glucose metabolism. These receptors have been proposed as therapeutic targets for metabolic diseases, and ISE (Iterative Stochastic Elimination) [116] has been developed, a tool that allows distinguishing agonist compounds from PPARs.

When the C1 complement component is over-activated, its regulation can be altered producing tissue damage that activates the complement system again, thus producing a circle of activations that perpetuates itself and produces ever greater damage. Treatments to inhibit C1 are expensive, so the search for cheaper inhibitors continues [117].

As mentioned, the development of new drugs is a complex and resource-intensive process. Therefore, the search for new clinical indications for existing drugs has become an alternative to accelerate and reduce the costs of the process. Thus, the term drug repositioning refers to the process of developing a compound for use in a pathology other than its current indication, taking advantage of the benefits of the abundance, variety and easy access to pharmaceutical products and biomedical data [118]. A promising approach to drug repositioning is to take advantage of machine learning algorithms to learn patterns in available drug-related biological data and link them to specific diseases to be treated [119], [120]. For example, indications for compounds against malaria, tuberculosis, and large cell carcinoma are already being reused for predicting protein interactions by calculating the accuracy by comparing similarity of interactions of approved drugs for other indications [121].

The WHO proposed a classification that assigns codes to compounds according to their therapeutic, pharmacological and chemical characteristics, as well as the sites of *in vivo* activity. The ability to predict the ATC codes of compounds can assist in the creation of high quality chemical libraries for compound detection and drug repositioning [30].

## 5 Trending in ML algorithms used in drug design

### 5.1 Naive Bayes

Generally speaking Machine Learning algorithms try to find the best hypothesis from a given data of interest. In particular for a classification problem the class for an unknown data sample. Bayesian classifiers assign the most likely class of each sample, according to the description given by the vector values of its variables. In its simplest version, the algorithm assumes that the variables are independent, that is, it facilitates the application of Bayes’ Theorem [122]. Although this assumption is unrealistic (not all variables are equally important), the family of classifiers that arises from the previous premise, known as NB (Naïve Bayes) obtain outstanding results, even though in some cases there are strong dependencies in their set of attributes. This algorithm describes a simple way to apply Bayes’ theorem to classification problems, and it is a simple, fast model that is capable of working with noisy data. It is able to learn from small data sets, which is an advantage although it does not suffer if the data volume is very high in terms of number of samples. It is not the ideal algorithm for high dimensionality problems with a high number of attributes since it uses frequency tables to extract knowledge from the data and treats each variable as categorical and, in case of working with numerical variables, it must perform some kind of transformation.

#### 5.1.1 Naive Bayes in drug discovery

This model has been used in drug discovery for the prediction of possible drug targets. Specifically, in [123] they developed a Bayesian model that integrates different data sources such as known side effects, or gene expression data, achieving a model with 90% accuracy on more than 2,000 molecules and also developing the experimental validation of the screening process. In [38] they predict molecules that are multi target with AUC 80% for treatment of VIH/HCV from data obtained from ChEMBL and generating for each of them two types of descriptors (MACCS and ECFP6) and validating the results by docking techniques. From 5125 known interactions with four different subtypes of proteins (enzymes, ion channels, GPCRs and nuclear receptors) obtained from KEGG and DrugBank and random interactions from STITCH in [40] they generated a model for the prediction of ligand-target interactions with an accuracy of 95%. In [30] they generate drug prediction models according to the ATM (Anatomical Therapeutic Chemical) system of the WHO using from STITCH and ChEMBL the data set used and calculating three different types of molecular descriptors (based on structure, interaction between compounds and interactions with similar targets) with an accuracy of 65%. In [31] they followed an experimental design based on machine learning and molecular docking for the prediction of possible inhibitors of Mttopo I, target protein for tuberculosis with AUC values of 74% and performing the in vitro validation of their computational results.

Furthermore, from the generation of different molecular descriptors of a set of compounds that damaged the liver in [39], they predict possible liver damage by traditional Chinese medicine with an accuracy of 72%. In [34] from 50 ChEMBL compounds, prediction models were generated with an AUC of 80% for inhibitors for IDO, calculating QSAR descriptors and validating the results by means of molecular docking techniques.

This type of model has also been used in [28] to predict the toxicity of chemical elements obtained from previous publications in Pubmed with an AUC higher than 80% and validating the best predictions in the laboratory. Or in [103] to predict PI3K inhibitors from 3D QSAR descriptors obtained from ChEMBL and BindigDB with AUC values of 97% and also with in vitro validation phase and by means of computational docking techniques.

### 5.2 Support vector machines

Vapnik introduced Support Vector Machines (SVM) in the late seventies [124]. They are one of the most widely used techniques because of their good performance and their ability to be generalized in high-dimensional domains, especially in bioinformatics [125], [126], [127]. In machine learning, sets of points in a given space are used to learn a way to deal with new observations. Kernel-based methods use those points to learn how similar the new observations are and to make a decision. Kernels code and measure the similarity between objects [128], [129], [130].

The base implementation works with two-class problems in which the data are separated by a hyperplane although there are implementations for regression or survival problems. Being n the dimension of the data, a hyperplane is an affine subspace of dimension n-1 that divides the space in two halves that corresponds to the entries of the two classes [130]. In the classification task, the goal of the SVM is to find the hyperplane that separates the positive examples from the negative ones. This hyperplane separates the positive examples from the negative ones, oriented in such a way that the distance between the border and the nearest data of each class is maximum; the nearest points are used to define the margins, known as support vectors [131]. You can see the concept of optimal hyperplanar.

Machine learning techniques, kernel-based methods and SVM more specifically, have proven to be exceptionally efficient in high dimensionality classification problems [132], [133] due to their ability to generalize into such spaces, as in the case of texture analysis.

Most complex datasets are not linearly separable, so SVM introduces the concept of kernel. A kernel function is a function that maps the input space to a higher dimension, where the data can be linearly separable. However, the inclusion of these kernels requires a new level of parameterization, where the kernel function and its parameters must be carefully selected.

In the case where the data is not linearly separable, one of the techniques used is the kernel trick. The idea is very simple and is based on what was mentioned in the previous two sections. The support vector machines look for the hyper plane that best separates the data, maximizing the generalization capacity of the model. If the data is not linearly separable, in an attempt to make it so, the initial input space can be mapped to a more dimensional space [134] (this is called the feature space). In this new space must be defined the scalar product, Hilbert’s space.

There are many kernels that can fulfill Mercer’s theorem [135]. A simple classification was proposed by Smola in her Doctoral Thesis, separating them into local and global kernels [136]. For local kernels only those data that are close to each other have an influence on the kernel values, while in global kernels all points, however distant from each other, have an influence on the kernel values. An example of a global kernel might be the polynomial and local kernel, the radial-based function kernel. Both will be presented below.

By far the most commonly used kernels are the linear kernel, the q-degree polynomial kernel, the radial base function kernel, the radial-basis function kernel (RBF), and the sigmoid function kernel.

#### 5.2.1 Support Vector Machines in drug discovery

One of the most widely used models in bioinformatics is the SVM because of its ability to deal with complex, non-linear, high-dimensional and noisy problems. They have been used in [137] with an accuracy value of 83.9% to classify drugs based on their categorization in KEGG. A new framework for the prediction of complex drug-target interaction networks from interaction matrices with F1 values of 80% has been proposed in [93]. It is also possible to predict the stability in human liver microsomes by calculating different molecular descriptors and chemical indices from 25 ChEMBL datasets with values close to 70% in validation [35].

An interesting new approach to predict the effect of a drug on a tumor line by obtaining information about the genes involved in the response of the drug in different tumors is the one followed in [101] from expression data (GEO). Specifically, they used three different types of tumors and by means of transfer learning they extracted information previously from different metadata of each tumor line with AUC values of 70%. Another clinical application in [112] is to generate predictive models of peripheral neuropathy after chemotherapy from QSAR and QSTR descriptors for the toxicity of 95 compounds approved by the FDA with an MCC of 80%. Or in the search for anti-malarial drugs with accurary of 90% in [80] and performing a final phase of experimental validation.

Using CORINA, it is possible to generate molecular descriptors to describe compounds and use this information to classify drugs of the nervous system and the degree of toxicity of the molecules, specifically in [115] 760 descriptors were used and those that contained more information were selected through a feature selection approach with an accuracy of 89%. SVMs were also used following a feature selection scheme on 3D QSAR descriptors in [138] for HDAC1 inhibitor prediction. In wrapper feature selection models combined with a metaheuristic as a genetic algorithm to predict from 2D QSAR the compounds (used 499) that inhibit the P-gp membrane protein (cancer) in [104] obtained good results validated later by molecular docking techniques.

An example of an advanced use of SVMs is Multiple Kernel Learning (MKL) where different linear combinations of SVMs with different parameters or kernels are generated to try to solve the same problem. This also allows to integrate different heterogeneous data sets available but at a cost of increasing the computational cost. In [97] they demonstrate how an MKL model is able to predict drug-target interactions with AUC values of 90% by integrating different available datasets of 1332 known interactions (interaction matrix, side effect, pathology or sequence).

However, in complex problems and used in conjunction with a metaheuristic such as a genetic algorithm can occur, with small datasets (136 drugs) the overtraining of the model with inadequate results in validation (PubChem) as in [117] for the prediction of C1 inhibitor candidates.

### 5.3 Tree-based models

Given the relative success of tree-based models in different fields because of the good results they usually achieve, there are many different implementations of tree-based models. One approach that stands out from the rest is that which makes use of decision trees. Specifically, a decision tree is a hierarchical structure composed of nodes and branches that join them. Although there are implementations for different types of problems such as regression, survival or outliers detection, the approach used for classification problems stands out. Within the hierarchical structure of a decision tree, root nodes, internal nodes or terminal nodes can be identified. The root node is usually represented at the top of the tree with no branches reaching it and with one or more branches starting from it. With respect to internal nodes, they have one branch that reaches them and two or more that start from them to the next level of the hierarchy. Finally, the terminal nodes do not have branches that start from them, as they are at the last level of the hierarchy.

Of the different algorithms that use decision trees, Random Forest stands out above all others [139]. It is a meta-algorithm that uses a set of decision trees (ensemble) to build a solution to the problem that it intends to solve through bagging and boosting approaches. The mode of operation assumes that RF grows the tree and the decision trees solve the problem individually, with each one contributing a vote in the resolution of the problem. As each tree may be exploring a different part of the solution space, RF must ultimately determine the overall solution to the problem and does so by considering the majority of votes.

As it is designed as a bagging approach what RF does is to separate the data set that is being analysed in 1/3 for validation and 2/3 for training in such a way that each individual decision tree is able to determine internally the generalisation error that it is making, this is known as out of bag error and the authors have demonstrated that it is equivalent to the error that the algorithm would make if it is used with cross validation. Finally, as each decision tree is being trained with different examples and attributes it is possible, from the OOB, to calculate an importance value of each of the attributes, being able to discard the rest and reducing the dimensionality of the problem. That is why this algorithm is particularly useful for very high dimensionality and noise problems.

#### 5.3.1 Random Forest in drug discovery

This is one of the most widely used algorithms in ML, regardless of the type of problem to be solved and, although it is not possible to identify a model as the best for any type of problem, RF is undoubtedly one of the best in terms of performance, speed and generalizability.

Using 211,888 compound-protein interactions from BindingDB in a mRMR (max relevance and min redundancy) dimensionality reduction scheme in [43] they were able to predict compound-protein interactions with an accuracy greater than 90% from the descriptors generated with Open Babel and the enrichment scores of each protein from GO and KEGG.

It is also possible to predict the interaction between a compound and a pathway from cMap data (it has 7056 microarray profiles of 5 cell lines, treated with 1309 different compounds). To do this, in [45] they calculated the molecular descriptors with RDKit and proposed a new tree-based model that uses the Relief algorithm for feature extraction and Graph-Based Semi-supervised Learning as a classifier with AUC results exceeding 90%. Moreover, it is possible to predict the interaction of a given drug with molecules in the plasma membrane of GPCR cells using PseAAC QSAR descriptors from 1860 GPCR-drug pairs with an accuracy of 87% as in [32]. In [105], prediction models were generated to test different antibodies on tumour cell lines quantifying proliferation and apoptosis levels from RF-selected variables to check those that best describe the phenotype induced by each antibody-dose.

It is also possible to calculate descriptors that are not molecular but proteochemometrics by pipeline pilot (512 descriptors) to predict possible inhibitors for SGLT1 in type II diabetes with a MCC value of 48% as in [33].

Moreover, the robustness of the model and its high performance in prediction tasks has made it possible to use it in the search for synergies with several drugs in different cell lines. In [110] they predict synergies between two drugs and a cell line using genomic information, drug targets and pharmacological information with a total of 583 drug combinations for 31 types of tumour cell lines. Based on gene expression and mutation data in cancer-related pathways, they identified tree-based models as the best predictors of synergy score. Even converting the problem into a ranking one they maintained F1 values of 95.4%. It is worth mentioning a joint effort of multiple researchers that emerges as a DREAM challenge in which from 11,576 experiments reported by AstraZeneca of 910 drug combinations on 85 molecularly characterised cancer cell lines (expression, copy number variation, methylation, mutations) [140], 160 international teams try to predict the best synergies between drug pairs and biomarkers for which different approaches were used: SVM, MKL, RF, decision trees or ANN. The winning team of the different prediction events used an RF.

### 5.4 Artificial Neural Networks

To be able to talk about ANN, we must first talk about the artificial neuron, which will be a functional element of the network which receives information from other elements and somehow processes them to end up providing an output that can be processed by other elements. As in nature, the artificial neurons can communicate with each other and the connections of the neurons are represented by weights that are no more than a value that tries to express the synaptic force of that connection between two neurons. When evaluating an artificial neuron or processing element the first thing to consider is the net value that represents the set of all the forces that they receive. Once the net value has been calculated, a trigger function is applied to determine the output of processing element. Based on the concept of an artificial neuron, several neurons can be interconnected to form a network where the outputs of one neuron can be the input of another neuron. It is necessary to understand that ANN needs to have input nodes which are the ones that obtain information from the outside; these neurons are said to be the input layer of the network. The network also needs output nodes, which are in the hidden layer, which transfer the ANN result. The rest of the nodes are known as hidden nodes that transmit information between neurons in the network and are grouped in one or several hidden layers.

Many researchers have defined specific network parameters such as its topology, the activation functions that modify the network output, in order to obtain different types of ANNs. It is important to mention that what is important about an ANN is not only the topology of the interconnection of the neurons and their activation functions, but a fundamental part is the relevance of each of the network connections. These values are obtained in a training phase, which depending on the type of network can be supervised, unsupervised and by reinforcement. It should be borne in mind that training a network is a time-consuming process. Obtaining the output of an ANN involves evaluating all the neurons that make up the network, and in training the process is iterative. For this reason, ANNs usually have a small number of neurons, which means that all the knowledge is in the tangle of connections without being able to know what action each part performs, which is why an ANN is considered to be a black box; the inputs to the network and the outputs it produces are known, but not what happens inside it.

However, there is a particular case of a neural network, Deep Learning. It usually has a very large number of layers of neurons connected to each other; although this does not really define deep learning. The concept behind Deep Learning is how information is processed, information is processed in a hierarchical way. In other words, in DL each layer of neurons tries to obtain a more meaningful representation of the data. The first layers extract a low level of characteristics, but as one goes deeper into the network, simple functions are combined to be able to represent more complex relationships.

The rise of ANN and DL models generally speaking arises from the computational explosion resulting from the widespread use of GPUs. This qualitative and quantitative leap meant a reduction of months/years in the training of complex models with thousands of internal layers in the hierarchy to minutes or seconds. Moreover, it meant moving from toy models to models that actually more closely resemble the biological hierarchical structure of the real human brain in terms of the number of neurons and layers. Unfortunately it is still necessary to wait to see equivalent models in number of connections (where the knowledge really lies) and the biological model.

However, despite the tremendous increase in the use of this type of technique, some of the shortcomings associated with the model remain unsolved: they are black boxes that issue an output from an input but do not explain how they reached that conclusion, they require very large volumes of data (partially solved with transfer learning and the possibility of reusing models trained for similar problems) and the choice of some of the existing model hierarchies for a new different problem is not easy.

#### 5.4.1 ANN in drug discovery

One of the first articles to use ANN for drug prediction is [141], trained ANN and tree-based algorithms from CMC (Comprehensive Medicinal Chemistry) and ACD (Available Chemicals directory) data, for drugs and non-drugs, respectively. For each of the compounds they used 1D descriptors that contained information about the whole molecule (molecular weight, number of hydrogen bonds, etc.) and 2D descriptors that contained information about the presence or absence of functional groups within the molecule. The best results were obtained with an ANN and both types of descriptors with an accuracy of 89%.

From 1003 chemicals from the Carinogenic Potency Database in [37], they predict the early carcinogenesis of compounds proposed to be drugs for which they calculate six different types of descriptors with a deep learning model and an accuracy of 86%. In the same way, it is possible to start an experimental phase in the laboratory to generate a set (2130 compounds) of possible new drugs of interest in cardiotoxicity and calculate with DRAGON 3456 descriptors of each of them and include the analysis in a feature selection scheme to end up with an AUC of 76%. A deep learning model with molecular descriptors of the compounds obtained from PubChem and Pfam proteins with an AUC above 95% was used from positive compound-protein interactions obtained from STITCH, taking randomly generated pairs as negative interactions.

Furthermore, as previously mentioned, it is possible to generate deep learning prediction models (Multi-channel PINN) using transfer learning to predict protein-linked interactions with three different types of descriptors and an AUC greater than 90% in Tox21 [26].

It is possible to work with information at different biological levels, for example in [87] they use deep learning to predict whether drug-protein binding is possible using transcriptomical data with 95% accuracy. In general, most of the works reviewed focus on the prediction of the function of a given drug, but it is still possible to advance in the field with regard to the prediction of interaction between ligand and protein with CNN, improving the results obtained with Vina, state-of-the-art software for Docking experiments from SMILES and FASTA. The rise of CNNs in image analysis has also enabled work in which, from different tumour cell lines, immunohistochemical images were generated for each of them and classification models were generated for normal and tumour lines. This approach [111] would therefore be useful in drug discovery. There are also works such as [58] in which this type of models are used to search for new molecules with the possibility of functioning as antiobiotics that have not yet reached the state of the art in generating descriptors based on graphs but which may represent an advance.

In general the most complex part of this ML model is obtaining datasets of sufficient size and finding the best hyperparameters for drug analysis [29]. It is even necessary to validate approaches such as dropout to establish whether they improve predictive performance in QSAR analyses studying drug-protein interaction as in [44]. As previously mentioned, the rise of these techniques means that they are applied to new domains and their performance needs to be carefully studied and the model adapted accordingly. Furthermore, in the field of drug discovery, machine learning systems are used to overcome the limitations of conventional drug discovery methods. It has even allowed the testing of drugs that were designed for a specific purpose for other purposes [78], techniques known as repositioning, of which we saw some examples developed with RF. Structure-based drug design has benefited from machine learning because it is much faster and more cost effective than traditional design [142].

## 6 Timeline of Machine Learning algorithms in drug discovery

It was in 1964 when Hansch et al. [143] proposed the Hansch equation. It was a linear regression model using physicochemical descriptors (such as the hydrophobicity parameter, the electronic parameter and the steric parameter), used to describe the 2D structure–activity relationship. Thus, using a predictive algorithm such as linear regression and molecular descriptors of the sequences, the field of study of QSAR began.

It was not until 1998, when Ajay et al. [141] introduced the concept of Drug-likeness. They built a model capable of predicting with high performance whether a molecule was a drug or not. They did this from 1D and 2D molecular descriptors. This paper was pioneering for the field of drug discovery based on ML algorithms.

**Fig. 6:**
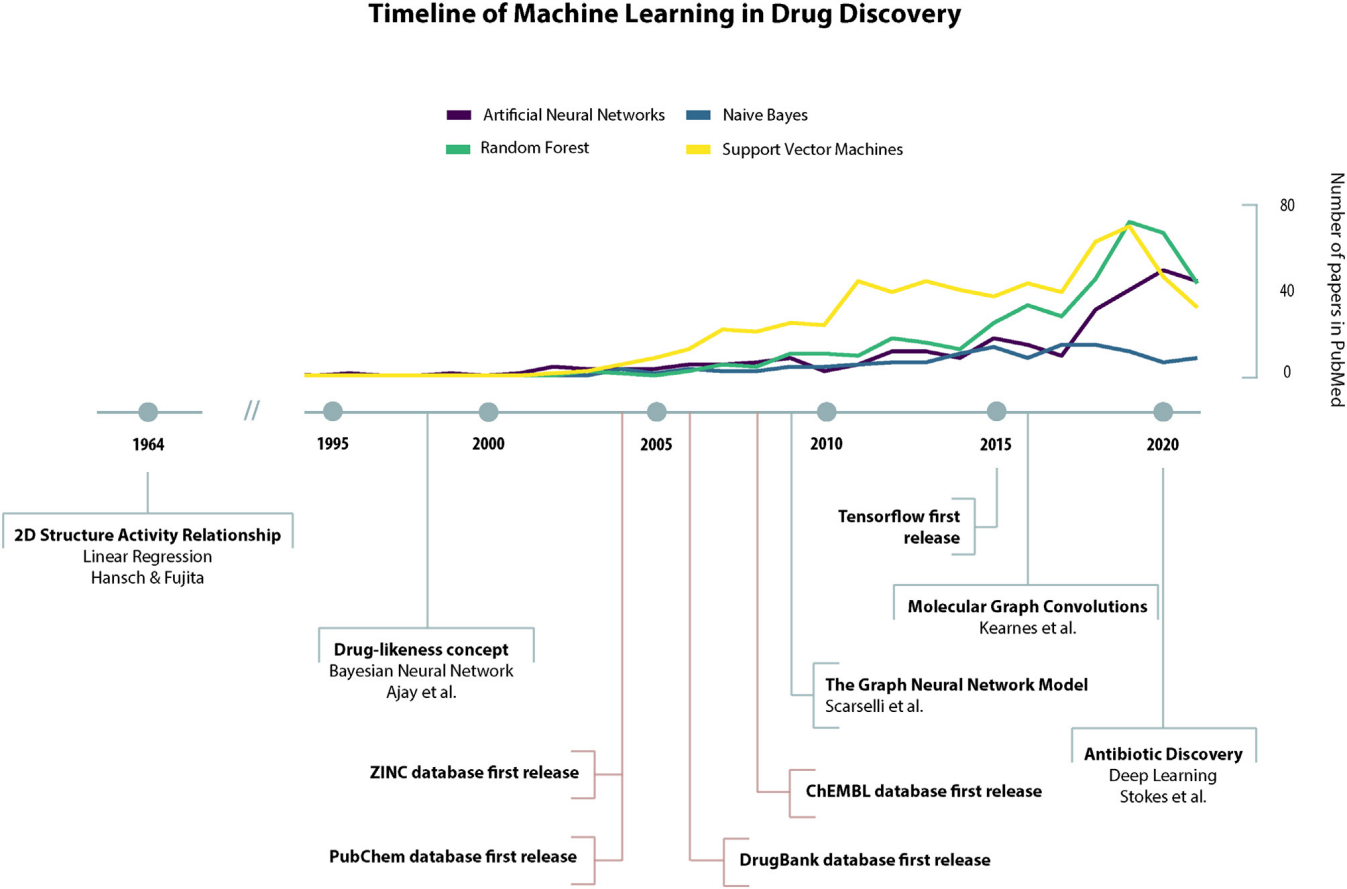
Timeline of Machine Learning main events in drug discovery field. The figure represents the main events of Machine Learning in drug discovery field. In addition, a line plot was drawn to show the paper counts along the time. Each algorithm is represented by a color line. The y-axis represents the number of papers published in PubMed.

There has been few published work based on ML in the field of drug discovery prior to the year 2000. The main reason has been the availability of data. With the advance of biotechnological and computational techniques, more and more molecule data have been generated and made available to the general public. In addition, large initiatives have developed public repositories where information on a large number of molecules is available in a standardized manner. It was in 2004 when the first version of two databases that will be of great importance for this field was released: PubChem and ZINC. Subsequently, in 2006 and 2008 DrugBank and ChEMBL were published, respectively. It was this fact, the availability of this large number of public databases, which allowed the development and training of new Machine Learning models to help in the screening of new drugs. To show this increase, Fig. 6 shows a history of the number of articles published in PubMed in this field. The search was stratified according to the different algorithms reviewed in this article. Specifically, a Boolean search was performed in PubMed with the following terms: algorithm name (’Artificial Neural Networks’, ’Support Vector Machines’, ’Naive Bayes’ or ’Random Forest’) & ’Drug Discovery’ term & 1964–2021 period. The results obtained from the four searches were downloaded and plotted together for comparison, as shown in the lineplot in Fig. 6.

As can be seen in the Fig. 6, from 2004–2008 there was a huge growth in the use of ML algorithms. Specifically, SVM has been by far the most widely used algorithm in recent years. A turning point in the use of neural networks was also observed from 2008 onwards. It was in this year that the Tensorflow library was released. From this moment on, the application of ANNs and especially the use of Deep Learning models boosted the number of publications in this field, until it became one of the most widely used algorithms. The wide variety of existing ANN topologies caused this growth. For example, The Graph Neural Network Model [51], published in 2009, opened up a new field of application in drug discovery. Until then, the vast majority of models used data from QSAR models. Thanks to these models, the inputs to the networks could be molecular graphs, for example, which also generated a large number of scientific publications. Subsequently, Molecular Graph Convolutions [58], another model that included the features of convolutional networks for the analysis of molecular graphs, was published in 2016. It was these models that were used in the work of Stokes et al. [78]. That work, published in 2020 in the journal Cell, demonstrated the capability of deep learning models in this field. They trained a deep learning model capable of predicting antibacterial activity. Subsequently, they made predictions on multiple chemical libraries, discovering a molecule, halicin, with antibacterial activity. This discovery was tested in wet lab, reinforcing the hypothesis established in the *in silico* experiments.

## 7 Conclusions

The latest advances in the design of new algorithms in the field of Artificial Intelligence have offered the opportunity to solve problems in different disciplines. In cheminformatics, and more specifically in drug discovery, the use of these models has greatly benefited the pharmaceutical industry. Previously, the only tool was the use of descriptors generated from the structure of small molecules or peptides. More recently, artificial neuron networks were adapted to model directly the molecules represented by graphs. Today, molecular descriptors are still widely used in the industry, but the rise of graph-based models is obtaining results that surpass the more conventional models in certain contexts. This fact is important because, although it is a developing branch of knowledge, it has very promising opportunities in the future, mainly due to its adaptability to the problems and molecular structures to be treated. On the other hand, as for the biological problems in which cheminformatics, and more specifically machine learning algorithms, have put their focus, it has been in the prediction of strong interactions for the search of new therapeutic targets. This observation is perfectly adapted in the context of precision medicine and to the requirements that this initiative presents.

Finally, and with the aim of highlighting this point, a joint effort must be made in the search for and use of standardized frameworks. This point is crucial for the rapid translation of academic results to the industry. Without a standardization of the processes and methodologies used, the results obtained cannot be extended to real clinical tasks. Therefore, the application of machine learning techniques must entail a robust design of the experiments for their replicability by different researchers. Throughout this review, this problem has been detected in the different articles reviewed. Therefore, in order to draw definitive conclusions, this aspect must be deeply influenced. However, the possibilities and advantages offered by ML techniques are immense within the context of precision medicine and drug discovery.

## Funding

This work was supported by the “Collaborative Project in Genomic Data Integration (CICLOGEN)” (Nos. PI17/01826, PI17/01561) funded by the Carlos III Health Institute from the Spanish National plan for Scientific and Technical Research and Innovation 2013–2016 and the European Regional Development Funds (FEDER)—“A way to build Europe.” and the General Directorate of Culture, Education and University Management of Xunta de Galicia (Ref. ED431D 2017/16), the “Galician Network for Colorectal Cancer Research” (Ref. ED431D 2017/23) and Competitive Reference Groups (Ref. ED431C 2018/49). The funding body did not have a role in the experimental design; data collection, analysis and interpretation; and writing of this manuscript. CITIC, as Research Center accredited by Galician University System, is funded by “Consellería de Cultura, Educación e Universidades from Xunta de Galicia”, supported in an 80% through ERDF Funds, ERDF Operational Programme Galicia 2014–2020, and the remaining 20% by “Secretaría Xeral de Universidades” (Grant ED431G 2019/01).

## CRediT authorship contribution statement

**Paula Carracedo-Reboredo:** Conceptualization, Methodology, Writing - original draft, Writing - review & editing. **Jose Li**ñ**ares-Blanco:** Conceptualization, Methodology, Writing - original draft, Writing - review & editing. **Nereida Rodríguez-Fernández:** Writing - original draft, Writing - review & editing. **Francisco Cedr**ó**n:** Supervision. **Francisco J. Novoa:** Supervision. **Adrian Carballal:** Supervision. **Victor Maojo:** Funding acquisition. **Alejandro Pazos:** Funding acquisition. **Carlos Fernandez-Lozano:** Conceptualization, Methodology, Project administration.

## Declaration of Competing Interest

The authors declare that they have no known competing financial interests or personal relationships that could have appeared to influence the work reported in this paper.

## References

[1] Collins F.S., Varmus H. (2015). A new initiative on precision medicine. New England J Med.

[2] Curtis C., Shah S.P., Chin S.-F., Turashvili G., Rueda O.M., Dunning M.J., Speed D., Lynch A.G., Samarajiwa S., Yuan Y. (2012). The genomic and transcriptomic architecture of 2,000 breast tumours reveals novel subgroups. Nature.

[3] Romond E.H., Perez E.A., Bryant J., Suman V.J., Geyer C.E., Davidson N.E., Tan-Chiu E., Martino S., Paik S., Kaufman P.A. (2005). Trastuzumab plus adjuvant chemotherapy for operable her2-positive breast cancer. N Engl J Med.

[4] Blanco J.L., Porto-Pazos A.B., Pazos A., Fernandez-Lozano C. (2018). Prediction of high anti-angiogenic activity peptides in silico using a generalized linear model and feature selection. Sci Rep.

[5] Munteanu C.R., Fernández-Blanco E., Seoane J.A., Izquierdo-Novo P., Angel Rodriguez-Fernandez J., Maria Prieto-Gonzalez J., Rabunal J.R., Pazos A. (2010). Drug discovery and design for complex diseases through qsar computational methods. Current Pharmaceutical Des.

[6] García I., Munteanu C.R., Fall Y., Gómez G., Uriarte E., González-Díaz H. (2009). Qsar and complex network study of the chiral hmgr inhibitor structural diversity. Bioorganic Med Chem.

[7] Liu Y., Tang S., Fernandez-Lozano C., Munteanu C.R., Pazos A., Yu Y.-Z., Tan Z., González-Díaz H. (2017). Experimental study and random forest prediction model of microbiome cell surface hydrophobicity. Expert Syst Appl.

[8] Riera-Fernández P., Munteanu C.R., Dorado J., Martin-Romalde R., Duardo-Sanchez A., Gonzalez-Diaz H. (2011). From chemical graphs in computer-aided drug design to general markov-galvez indices of drug-target, proteome, drug-parasitic disease, technological, and social-legal networks. Current Computer-aided Drug Des.

[9] Shirvani P., Fassihi A. (2020). Molecular modelling study on pyrrolo [2, 3-b] pyridine derivatives as c-met kinase inhibitors, a combined approach using molecular docking, 3d-qsar modelling and molecular dynamics simulation. Mol Simul.

[10] B. Suay-Garcia, J.I. Bueso-Bordils, A. Falcó, M.T. Pérez-Gracia, G. Antón-Fos, P. Alemán-López, Quantitative structure–activity relationship methods in the discovery and development of antibacterials, Wiley Interdisciplinary Reviews: Computational Molecular Science e1472.

[11] Fernandez-Lozano C., Gestal M., Munteanu C.R., Dorado J., Pazos A. (2016). A methodology for the design of experiments in computational intelligence with multiple regression models. PeerJ.

[12] D.S. Wishart, Y.D. Feunang, A.C. Guo, E.J. Lo, A. Marcu, J.R. Grant, T. Sajed, D. Johnson, C. Li, Z. Sayeeda, et al., Drugbank 5.0: a major update to the drugbank database for 2018, Nucleic acids research 46 (D1) (2018) D1074–D1082. 10.1093/nar/gkx1037 PMC5753335 29126136

[13] Kim S., Chen J., Cheng T., Gindulyte A., He J., He S., Li Q., Shoemaker B.A., Thiessen P.A., Yu B. (2019). Pubchem 2019 update: improved access to chemical data. Nucleic Acids Res.

[14] Gaulton A., Bellis L.J., Bento A.P., Chambers J., Davies M., Hersey A., Light Y., McGlinchey S., Michalovich D., Al-Lazikani B. (2012). Chembl: a large-scale bioactivity database for drug discovery. Nucleic Acids Res.

[15] Sterling T., Irwin J.J. (2015). Zinc 15–ligand discovery for everyone. J Chem Inform Modeling.

[16] Saeys Y., Inza I., Larrañaga P. (2007). A review of feature selection techniques in bioinformatics. Bioinformatics.

[17] Gramatica P., Sangion A. (2016). A historical excursus on the statistical validation parameters for qsar models: a clarification concerning metrics and terminology. J Chem Inform Modeling.

[18] Stephenson N., Shane E., Chase J., Rowland J., Ries D., Justice N., Zhang J., Chan L., Cao R. (2019). Survey of machine learning techniques in drug discovery. Current Drug Metabolism.

[19] Vamathevan J., Clark D., Czodrowski P., Dunham I., Ferran E., Lee G., Li B., Madabhushi A., Shah P., Spitzer M. (2019). Applications of machine learning in drug discovery and development. Nat Rev Drug Discovery.

[20] Gramatica P. (2020). Principles of qsar modeling: comments and suggestions from personal experience. Int J Quantitative Structure-Property Relationships (IJQSPR).

[21] Cronin M.T., Schultz T.W. (2003). Pitfalls in qsar. J Mol Struct (Thoechem).

[22] Cramer R.D., Patterson D.E., Bunce J.D. (1988). Comparative molecular field analysis (comfa). 1. effect of shape on binding of steroids to carrier proteins. J Am Chem Soc.

[23] Valdés-Martiní J.R., Marrero-Ponce Y., García-Jacas C.R., Martinez-Mayorga K., Barigye S.J., d’Almeida Y.S.V., Pérez-Giménez F., Morell C.A. (2017). Qubils-mas, open source multi-platform software for atom-and bond-based topological (2d) and chiral (2.5 d) algebraic molecular descriptors computations. J Cheminformatics.

[24] Fourches D., Ash J. (2019). 4d-quantitative structure–activity relationship modeling: making a comeback. Expert Opinion Drug Discovery.

[25] Wang T., Yuan X.-S., Wu M.-B., Lin J.-P., Yang L.-R. (2017). The advancement of multidimensional qsar for novel drug discovery-where are we headed?. Expert Opinion Drug Discovery.

[26] Lee M., Kim H., Joe H., Kim H.-G. (2019). Multi-channel pinn: investigating scalable and transferable neural networks for drug discovery. J Cheminformatics.

[27] Lee H.-M., Yu M.-S., Kazmi S.R., Oh S.Y., Rhee K.-H., Bae M.-A., Lee B.H., Shin D.-S., Oh K.-S., Ceong H. (2019). Computational determination of herg-related cardiotoxicity of drug candidates. BMC Bioinformatics.

[28] Perryman A.L., Patel J.S., Russo R., Singleton E., Connell N., Ekins S., Freundlich J.S. (2018). Naive bayesian models for vero cell cytotoxicity. Pharmaceut Res.

[29] Koutsoukas A., Monaghan K.J., Li X., Huan J. (2017). Deep-learning: investigating deep neural networks hyper-parameters and comparison of performance to shallow methods for modeling bioactivity data. J Cheminformatics.

[30] Olson T., Singh R. (2017). Predicting anatomic therapeutic chemical classification codes using tiered learning. BMC Bioinformatics.

[31] Ekins S., Godbole A.A., Kéri G., Orfi L., Pato J., Bhat R.S., Verma R., Bradley E.K., Nagaraja V. (2017). Machine learning and docking models for mycobacterium tuberculosis topoisomerase i. Tuberculosis.

[32] Hu J., Li Y., Yang J.-Y., Shen H.-B., Yu D.-J. (2016). Gpcr–drug interactions prediction using random forest with drug-association-matrix-based post-processing procedure. Comput Biology Chemistry.

[33] L. Burggraaff, P. Oranje, R. Gouka, P. van der Pijl, M. Geldof, H.W. van Vlijmen, A.P. IJzerman, G.J. van Westen, Identification of novel small molecule inhibitors for solute carrier sglt1 using proteochemometric modeling, Journal of cheminformatics 11 (1) (2019) 15 10.1186/s13321-019-0337-8 PMC6689890 30767155

[34] Zhang H., Liu W., Liu Z., Ju Y., Xu M., Zhang Y., Wu X., Gu Q., Wang Z., Xu J. (2018). Discovery of indoleamine 2, 3-dioxygenase inhibitors using machine learning based virtual screening. MedChemComm.

[35] Aliagas I., Gobbi A., Heffron T., Lee M.-L., Ortwine D.F., Zak M., Khojasteh S.C. (2015). A probabilistic method to report predictions from a human liver microsomes stability qsar model: a practical tool for drug discovery. J Comput Aided Mol Des.

[36] Ayed M., Lim H., Xie L. (2019). Biological representation of chemicals using latent target interaction profile. BMC Bioinformatics.

[37] Wang Y.-W., Huang L., Jiang S.-W., Li K., Zou J., Yang S.-Y. (2020). Capscarcino: A novel sparse data deep learning tool for predicting carcinogens. Food Chem Toxicol.

[38] Wei Y., Li W., Du T., Hong Z., Lin J. (2019). Targeting hiv/hcv coinfection using a machine learning-based multiple quantitative structure-activity relationships (multiple qsar) method. Int J Molecular Sci.

[39] He S., Zhang X., Lu S., Zhu T., Sun G., Sun X. (2019). A computational toxicology approach to screen the hepatotoxic ingredients in traditional chinese medicines: Polygonum multiflorum thunb as a case study. Biomolecules.

[40] Li L., Koh C.C., Reker D., Brown J., Wang H., Lee N.K., Liow H.-H., Dai H., Fan H.-M., Chen L. (2019). Predicting protein-ligand interactions based on bow-pharmacological space and bayesian additive regression trees. Sci Rep.

[41] Di P., Yin Y., Jiang C., Cai Y., Li W., Tang Y., Liu G. (2019). Prediction of the skin sensitising potential and potency of compounds via mechanism-based binary and ternary classification models. Toxicol In Vitro.

[42] Huang G., Li J., Zhao C. (2018). Computational prediction and analysis of associations between small molecules and binding-associated s-nitrosylation sites. Molecules.

[43] Chen L., Zhang Y.-H., Zheng M., Huang T., Cai Y.-D. (2016). Identification of compound–protein interactions through the analysis of gene ontology, kegg enrichment for proteins and molecular fragments of compounds. Mol Genet Genomics.

[44] Mendenhall J., Meiler J. (2016). Improving quantitative structure–activity relationship models using artificial neural networks trained with dropout. J Computer-aided Mol Design.

[45] Song M., Jiang Z. (2015). Inferring association between compound and pathway with an improved ensemble learning method. Mol Inform.

[46] Tian K., Shao M., Wang Y., Guan J., Zhou S. (2016). Boosting compound-protein interaction prediction by deep learning. Methods.

[47] Dong J., Yao Z.-J., Zhu M.-F., Wang N.-N., Lu B., Chen A.F., Lu A.-P., Miao H., Zeng W.-B., Cao D.-S. (2017). Chemsar: an online pipelining platform for molecular sar modeling. J Cheminformatics.

[48] Cai C., Wu Q., Luo Y., Ma H., Shen J., Zhang Y., Yang L., Chen Y., Wen Z., Wang Q. (2017). In silico prediction of rock ii inhibitors by different classification approaches. Mol Diversity.

[49] Sun M., Zhao S., Gilvary C., Elemento O., Zhou J., Wang F. (2020). Graph convolutional networks for computational drug development and discovery. Briefings Bioinformatics.

[50] Na G.S., Kim H.W., Chang H. (2020). Costless performance improvement in machine learning for graph-based molecular analysis. J Chem Inf Model.

[51] Scarselli F., Gori M., Tsoi A.C., Hagenbuchner M., Monfardini G. (2008). The graph neural network model. IEEE Trans Neural Networks.

[52] D.K. Duvenaud, D. Maclaurin, J. Iparraguirre, R. Bombarell, T. Hirzel, A. Aspuru-Guzik, R.P. Adams, Convolutional networks on graphs for learning molecular fingerprints, in: Advances in neural information processing systems, 2015, pp. 2224–2232.

[53] J. Bruna, W. Zaremba, A. Szlam, Y. LeCun, Spectral networks and locally connected networks on graphs, arXiv preprint arXiv:1312.6203.

[54] Masci J., Boscaini D., Bronstein M., Vandergheynst P. (2015). Proceedings of the IEEE international conference on computer vision workshops.

[55] Coley C.W., Jin W., Rogers L., Jamison T.F., Jaakkola T.S., Green W.H., Barzilay R., Jensen K.F. (2019). A graph-convolutional neural network model for the prediction of chemical reactivity. Chem Sci.

[56] Merkwirth C., Lengauer T. (2005). Automatic generation of complementary descriptors with molecular graph networks. J Chem Inform Modeling.

[57] Micheli A. (2009). Neural network for graphs: A contextual constructive approach. IEEE Trans Neural Networks.

[58] Kearnes S., McCloskey K., Berndl M., Pande V., Riley P. (2016). Molecular graph convolutions: moving beyond fingerprints. J Computer-aided Mol Design.

[59] Na G.S., Chang H., Kim H.W. (2020). Machine-guided representation for accurate graph-based molecular machine learning. PCCP.

[60] Jippo H., Matsuo T., Kikuchi R., Fukuda D., Matsuura A., Ohfuchi M. (2020). Graph classification of molecules using force field atom and bond types. Mol Inform.

[61] Khemchandani Y., O’Hagan S., Samanta S., Swainston N., Roberts T.J., Bollegala D., Kell D.B. (2020). Deepgraphmolgen, a multi-objective, computational strategy for generating molecules with desirable properties: a graph convolution and reinforcement learning approach. J Cheminformatics.

[62] Ye S., Liang J., Liu R., Zhu X. (2020). Symmetrical graph neural network for quantum chemistry with dual real and momenta space. J Phys Chem A.

[63] X. Sun, N.J. Krakauer, A. Politowicz, W.-T. Chen, Q. Li, Z. Li, X. Shao, A. Sunaryo, M. Shen, J. Wang, et al., Assessing graph-based deep learning models for predicting flash point, Molecular Informatics. 10.1002/minf.201900101 32077235

[64] Z. Xiong, D. Wang, X. Liu, F. Zhong, X. Wan, X. Li, Z. Li, X. Luo, K. Chen, H. Jiang, et al., Pushing the boundaries of molecular representation for drug discovery with the graph attention mechanism, Journal of Medicinal Chemistry. 10.1021/acs.jmedchem.9b00959 31408336

[65] Tian S., Wang J., Li Y., Li D., Xu L., Hou T. (2015). The application of in silico drug-likeness predictions in pharmaceutical research. Adv Drug Delivery Rev.

[66] Burton M.E. (2006).

[67] Liu K., Sun X., Jia L., Ma J., Xing H., Wu J., Gao H., Sun Y., Boulnois F., Fan J. (2019). Chemi-net: a molecular graph convolutional network for accurate drug property prediction. Int J Mol Sci.

[68] A.H. Göller, L. Kuhnke, F. Montanari, A. Bonin, S. Schneckener, A. Ter Laak, J. Wichard, M. Lobell, A. Hillisch, Bayer’s in silico admet platform: A journey of machine learning over the past two decades, Drug Discovery Today. 10.1016/j.drudis.2020.07.001 32652309

[69] Prasad S., Brooks B.R. (2020). A deep learning approach for the blind logp prediction in sampl6 challenge. J Comput Aided Mol Des.

[70] Lu J., Lu D., Zhang X., Bi Y., Cheng K., Zheng M., Luo X. (2016). Estimation of elimination half-lives of organic chemicals in humans using gradient boosting machine. Biochimica et Biophysica Acta (BBA)-General Subjects.

[71] Raja K., Patrick M., Elder J.T., Tsoi L.C. (2017). Machine learning workflow to enhance predictions of adverse drug reactions (adrs) through drug-gene interactions: application to drugs for cutaneous diseases. Sci Rep.

[72] Li H., Tang B., Chen Q., Chen K., Wang X., Wang B., Wang Z. (2016). Hitsz_cdr: an end-to-end chemical and disease relation extraction system for biocreative v. Database.

[73] Hu B., Wang H., Wang L., Yuan W. (2018). Adverse drug reaction predictions using stacking deep heterogeneous information network embedding approach. Molecules.

[74] Zhang W., Liu F., Luo L., Zhang J. (2015). Predicting drug side effects by multi-label learning and ensemble learning. BMC Bioinformatics.

[75] Lu J., Lu D., Fu Z., Zheng M., Luo X. (2018). Computational Systems Biology.

[76] Onguéné P.A., Simoben C.V., Fotso G.W., Andrae-Marobela K., Khalid S.A., Ngadjui B.T., Mbaze L.M., Ntie-Kang F. (2018). In silico toxicity profiling of natural product compound libraries from african flora with anti-malarial and anti-hiv properties. Comput Biology Chemistry.

[77] Serafim M.S.M., Kronenberger T., Oliveira P.R., Poso A., Honorio K.M., Mota B.E.F., Maltarollo V.G. (2020). The application of machine learning techniques to innovative antibacterial discovery and development. Expert Opin Drug Discov.

[78] Stokes J.M., Yang K., Swanson K., Jin W., Cubillos-Ruiz A., Donghia N.M., MacNair C.R., French S., Carfrae L.A., Bloom-Ackerman Z. (2020). A deep learning approach to antibiotic discovery. Cell.

[79] KalantarMotamedi Y., Eastman R.T., Guha R., Bender A. (2018). A systematic and prospectively validated approach for identifying synergistic drug combinations against malaria. Malaria J.

[80] Viira B., Gendron T., Lanfranchi D.A., Cojean S., Horvath D., Marcou G., Varnek A., Maes L., Maran U., Loiseau P.M. (2016). In silico mining for antimalarial structure-activity knowledge and discovery of novel antimalarial curcuminoids. Molecules.

[81] Schuler J., Hudson M.L., Schwartz D., Samudrala R. (2017). A systematic review of computational drug discovery, development, and repurposing for ebola virus disease treatment. Molecules.

[82] A. Alimadadi, S. Aryal, I. Manandhar, P.B. Munroe, B. Joe, X. Cheng, Artificial intelligence and machine learning to fight covid-19 (2020). 10.1152/physiolgenomics.00029.2020 PMC7191426 32216577

[83] De P., Bhayye S., Kumar V., Roy K. (2020). In silico modeling for quick prediction of inhibitory activity against 3clpro enzyme in sars cov diseases. J Biomol Struct Dyn.

[84] Kumar V., Roy K. (2020). Development of a simple, interpretable and easily transferable qsar model for quick screening antiviral databases in search of novel 3c-like protease (3clpro) enzyme inhibitors against sars-cov diseases. SAR QSAR Environ Res.

[85] Brito-Sánchez Y., Marrero-Ponce Y., Barigye S.J., Yaber-Goenaga I., Morell Perez C., Le-Thi-Thu H., Cherkasov A. (2015). Towards better bbb passage prediction using an extensive and curated data set. Mol Inform.

[86] Sharma A., Rani R. (2018). Be-dti’: Ensemble framework for drug target interaction prediction using dimensionality reduction and active learning. Computer Methods Programs Biomed.

[87] Xie L., He S., Song X., Bo X., Zhang Z. (2018). Deep learning-based transcriptome data classification for drug-target interaction prediction. BMC Genomics.

[88] Chen R., Liu X., Jin S., Lin J., Liu J. (2018). Machine learning for drug-target interaction prediction. Molecules.

[89] Deng J., Yuan Q., Mamitsuka H., Zhu S. (2018). Data Mining for Systems Biology.

[90] Sunseri J., King J.E., Francoeur P.G., Koes D.R. (2019). Convolutional neural network scoring and minimization in the d3r 2017 community challenge. J Computer-aided Mol Design.

[91] J.B. Cross, Methods for virtual screening of gpcr targets: Approaches and challenges, in: Computational Methods for GPCR Drug Discovery, Springer, 2018, pp. 233–264. 10.1007/978-1-4939-7465-8_11 29188566

[92] Chen Y., Argentinis J.E., Weber G. (2016). Ibm watson: how cognitive computing can be applied to big data challenges in life sciences research. Clinical Therapeutics.

[93] Fu G., Ding Y., Seal A., Chen B., Sun Y., Bolton E. (2016). Predicting drug target interactions using meta-path-based semantic network analysis. BMC Bioinformatics.

[94] Kana O., Brylinski M. (2019). Elucidating the druggability of the human proteome with efindsite. J Computer-aided Mol Design.

[95] Hu B., Kun Kuang Z., Feng S.-Y., Wang D., He S.-B., Xin Kong D. (2016). Supplementary materials: Three-dimensional biologically relevant spectrum (brs-3d): Shape similarity profile based on pdb ligands as molecular descriptor. Molecules.

[96] Lu C., Liu Z., Zhang E., He F., Ma Z., Wang H. (2019). Mpls-pred: Predicting membrane protein-ligand binding sites using hybrid sequence-based features and ligand-specific models. Int J Mol Sci.

[97] Yan X.-Y., Zhang S.-W., He C.-R. (2019). Prediction of drug-target interaction by integrating diverse heterogeneous information source with multiple kernel learning and clustering methods. Comput Biology Chemistry.

[98] Li Q., Watkins M., Robinson S.D., Safavi-Hemami H., Yandell M. (2018). Discovery of novel conotoxin candidates using machine learning. Toxins.

[99] Plante A., Shore D.M., Morra G., Khelashvili G., Weinstein H. (2019). A machine learning approach for the discovery of ligand-specific functional mechanisms of gpcrs. Molecules.

[100] Xing J., Zhang R., Jiang X., Hu T., Wang X., Qiao G., Wang J., Yang F., Luo X., Chen K. (2019). Rational design of 5-((1h-imidazol-1-yl) methyl) quinolin-8-ol derivatives as novel bromodomain-containing protein 4 inhibitors. Eur J Med Chem.

[101] Turki T., Wang J.T. (2019). Clinical intelligence: New machine learning techniques for predicting clinical drug response. Computers Biol Med.

[102] Fan C., Huang Y. (2017). Identification of novel potential scaffold for class i hdacs inhibition: An in-silico protocol based on virtual screening, molecular dynamics, mathematical analysis and machine learning. Biochem Biophys Res Commun.

[103] Yu M., Gu Q., Xu J. (2018). Discovering new pi3k*α*) inhibitors with a strategy of combining ligand-based and structure-based virtual screening. J Computer-aided Mol Design.

[104] Ngo T.-D., Tran T.-D., Le M.-T., Thai K.-M. (2016). Computational predictive models for p-glycoprotein inhibition of in-house chalcone derivatives and drug-bank compounds. Mol Diversity.

[105] Sandercock A.M., Rust S., Guillard S., Sachsenmeier K.F., Holoweckyj N., Hay C., Flynn M., Huang Q., Yan K., Herpers B. (2015). Identification of anti-tumour biologics using primary tumour models, 3-d phenotypic screening and image-based multi-parametric profiling. Mol Cancer.

[106] Guo Q., Luo Y., Zhai S., Jiang Z., Zhao C., Xu J., Wang L. (2019). Discovery, biological evaluation, structure–activity relationships and mechanism of action of pyrazolo [3, 4-b] pyridin-6-one derivatives as a new class of anticancer agents. Organic Biomol Chem.

[107] Ung P.M.-U., Rahman R., Schlessinger A. (2019). Redefining the protein kinase conformational space with machine learning. Biophys J.

[108] Gautam P., Jaiswal A., Aittokallio T., Al-Ali H., Wennerberg K. (2019). Phenotypic screening combined with machine learning for efficient identification of breast cancer-selective therapeutic targets. Cell Chem Biol.

[109] Valdebenito S., Lou E., Baldoni J., Okafo G., Eugenin E. (2018). The novel roles of connexin channels and tunneling nanotubes in cancer pathogenesis. Int J Mol Sci.

[110] Jeon M., Kim S., Park S., Lee H., Kang J. (2018). In silico drug combination discovery for personalized cancer therapy. BMC Syst Biol.

[111] Radhakrishnan A., Damodaran K., Soylemezoglu A.C., Uhler C., Shivashankar G. (2017). Machine learning for nuclear mechano-morphometric biomarkers in cancer diagnosis. Sci Rep.

[112] Bloomingdale P., Mager D.E. (2019). Machine learning models for the prediction of chemotherapy-induced peripheral neuropathy. Pharm Res.

[113] Romeo-Guitart D., Forés J., Herrando-Grabulosa M., Valls R., Leiva-Rodríguez T., Galea E., González-Pérez F., Navarro X., Petegnief V., Bosch A. (2018). Neuroprotective drug for nerve trauma revealed using artificial intelligence. Sci Rep.

[114] Luo M., Reid T.-E., Simon Wang X. (2015). Discovery of natural product-derived 5-ht1a receptor binders by cheminfomatics modeling of known binders, high throughput screening and experimental validation. Combinatorial Chem High Throughput Screening.

[115] Onay A., Onay M., Abul O. (2017). Classification of nervous system withdrawn and approved drugs with toxprint features via machine learning strategies. Comput Methods Programs Biomed.

[116] Da’adoosh B., Marcus D., Rayan A., King F., Che J., Goldblum A. (2019). Discovering highly selective and diverse ppar-delta agonists by ligand based machine learning and structural modeling. Sci Rep.

[117] Chen J.J., Schmucker L.N., Visco D.P. (2018). Pharmaceutical machine learning: Virtual high-throughput screens identifying promising and economical small molecule inhibitors of complement factor c1s. Biomolecules.

[118] Nam Y., Kim M., Chang H.-S., Shin H. (2019). Drug repurposing with network reinforcement. BMC Bioinformatics.

[119] K. Zhao, H.-C. So, Using drug expression profiles and machine learning approach for drug repurposing, in: Computational methods for drug repurposing, Springer, 2019, pp. 219–237. 10.1007/978-1-4939-8955-3_13 30547445

[120] Y. Wang, J. Yella, A.G. Jegga, Transcriptomic data mining and repurposing for computational drug discovery, in: Computational Methods for Drug Repurposing, Springer, 2019, pp. 73–95. 10.1007/978-1-4939-8955-3_5 30547437

[121] Mangione W., Samudrala R. (2019). Identifying protein features responsible for improved drug repurposing accuracies using the cando platform: Implications for drug design. Molecules.

[122] T. Bayes, Lii. an essay towards solving a problem in the doctrine of chances. by the late rev. mr. bayes, frs communicated by mr. price, in a letter to john canton, amfr s, Philosophical transactions of the Royal Society of London (53) (1763) 370–418.

[123] Madhukar N.S., Khade P.K., Huang L., Gayvert K., Galletti G., Stogniew M., Allen J.E., Giannakakou P., Elemento O. (2019). A bayesian machine learning approach for drug target identification using diverse data types. Nature Commun.

[124] Vapnik V. (2006).

[125] Schölkopf B., Tsuda K., Vert J.-P. (2004).

[126] Fernandez-Lozano C., Gestal M., Pedreira-Souto N., Postelnicu L., Dorado J., Robert Munteanu C. (2013). Kernel-based feature selection techniques for transport proteins based on star graph topological indices. Current Topics Med Chem.

[127] Fernandez-Lozano C., Gestal M., González-Díaz H., Dorado J., Pazos A., Munteanu C.R. (2014). Markov mean properties for cell death-related protein classification. J Theor Biol.

[128] Campbell C., Ying Y. (2011). Learning with support vector machines. Synthesis Lectures Artif Intell Mach Learn.

[129] Shawe-Taylor J., Cristianini N. (2004).

[130] Cristianini N., Shawe-Taylor J. (2000).

[131] Burges C.J. (1998). A tutorial on support vector machines for pattern recognition. Data Mining Knowledge Discovery.

[132] Chapelle O., Haffner P., Vapnik V.N. (1999). Support vector machines for histogram-based image classification. IEEE Trans Neural Networks.

[133] Moulin L., Da Silva A.A., El-Sharkawi M., Marks R.J. (2004). Support vector machines for transient stability analysis of large-scale power systems. IEEE Trans Power Syst.

[134] Scholkopf B., Mika S., Burges C.J., Knirsch P., Muller K.-R., Ratsch G., Smola A.J. (1999). Input space versus feature space in kernel-based methods. IEEE Trans Neural Networks.

[135] Mercer J. (1909). Xvi. functions of positive and negative type, and their connection the theory of integral equations. Philos Trans Royal Society London. Series A.

[136] Smola A.J., Schölkopf B. (2004). A tutorial on support vector regression. Stat Comput.

[137] Che J., Chen L., Guo Z.-H., Wang S. (2020). Drug target group prediction with multiple drug networks. Comb Chem High Throughput Screening.

[138] Hu B., Kuang Z.-K., Feng S.-Y., Wang D., He S.-B., Kong D.-X. (2016). Three-dimensional biologically relevant spectrum (brs-3d): shape similarity profile based on pdb ligands as molecular descriptors. Molecules.

[139] Breiman L. (2001). Random forests. Mach Learn.

[140] Menden M.P., Wang D., Mason M.J., Szalai B., Bulusu K.C., Guan Y., Yu T., Kang J., Jeon M., Wolfinger R. (2019). Community assessment to advance computational prediction of cancer drug combinations in a pharmacogenomic screen. Nature Commun.

[141] Ajay W.P., Walters M.A. (1998). Murcko, Can we learn to distinguish between drug-like and nondrug-like molecules?. J Med Chem.

[142] Batool M., Ahmad B., Choi S. (2019). A structure-based drug discovery paradigm. Int J Mol Sci.

[143] Hansch C., Fujita T. (1964). p-σ-π analysis. a method for the correlation of biological activity and chemical structure. J Am Chem Soc.

